# Multidomain intervention for delaying aging in community-dwelling older adults (MIDA): study design and protocol

**DOI:** 10.1080/07853890.2025.2496409

**Published:** 2025-04-29

**Authors:** Siyang Lin, Fang Wang, Min Huang, Jingyi Chen, Xinye Jiang, Qiaowei Li, Yin Yuan, Feng Huang, Pengli Zhu

**Affiliations:** a Fuzhou University Affiliated Provincial Hospital, Fuzhou, China; b Shengli Clinical Medical College of Fujian Medical University, Fuzhou, China; c Fujian Provincial Institute of Clinical Geriatrics, Fuzhou, China; d Fujian Provincial Key Laboratory of Geriatrics, Fuzhou, China; e Fujian Provincial Center for Geriatrics, Fuzhou, China; f Department of Nursing, Fuzhou University Affiliated Provincial Hospital, Fuzhou, China; g College of Nursing, Fujian University of Traditional Chinese Medicine, Fuzhou, China; h Department of Geriatric Medicine, 900TH Hospital of Joint Logistics Support Force, Fuzhou, China; i College of Integrative Medicine, Fujian University of Traditional Chinese Medicine, Fuzhou, China

**Keywords:** Multidomain intervention, DNA methylation age, biological age, frailty, intrinsic capacity

## Abstract

**Background:**

The exploration of interventions to delay aging is an emerging topic that promotes healthy aging. The multidomain intervention has the potential to be applied in the field of aging because it concentrates on the functional ability of older adults. There is currently no literature reporting on a multidomain intervention involving cognition, exercise and nutrition for delaying aging.

**Methods:**

The Multidomain Intervention for Delaying Aging in Community-dwelling Older Adults (MIDA) is a Zelen-design randomized controlled trial with a 6-month intervention duration. The multidomain intervention comprises cognitive training, exercise training, and nutritional guidance, delivered through both group sessions and individual family interventions. A total of 248 participants aged 60 to 85 years will be randomized to the intervention group or control group and followed up for 12 months. The primary outcome is the change in epigenetic age acceleration and pace of aging following the multidomain intervention. The secondary outcomes are the changes in frailty score and intrinsic capacity Z-score. Other outcomes include physical functions, body composition, aging biomarkers, inflammatory markers, haematology and biochemistry parameters, and lifestyle factors.

**Conclusions:**

This study will explore the effects of the multidomain intervention on delaying aging in community-dwelling older adults. We aim to introduce a new approach to delaying aging and offer a practical multidomain intervention strategy for healthcare institutions.

## Introduction

In recent years, studies on aging interventions have mainly focused on singular approaches such as anti-aging drugs, calorie restriction diets, and exercise interventions [1]. However, contemporary geriatricians advocate for a holistic intervention model that integrates multiple intervention strategies. This transformation is rooted in the understanding that promoting health is a person-centered process that encompasses the entire organism, rather than single disease or specific organ functions [2]. Older populations often have multiple risk factors and complex health conditions, making single-type interventions insufficient to address all health issues. The multidomain approach to aging intervention not only allows each intervention component to serve different health goals but also results in better delayed aging effects compared to single-type interventions [2,3].

Multidomain intervention, as a concept combining two or more intervention modes, has currently gained widespread attention in the field of geriatric medicine [4]. Aging is a multifactorial process involving declines in physical, cognitive, and nutritional aspects. Therefore, in our multidomain intervention strategies, we concentrate on three intervention methods: cognitive intervention, exercise intervention, and nutritional intervention. As they are used as single-type interventions, they can respectively bring about positive improvements in specific diseases or geriatric syndromes among the older population. Firstly, exercise intervention can not only prevent cardiovascular diseases, diabetes, and obesity, but also improve muscle function, mental health, and quality of life, and reduce all-cause mortality [5]. Meanwhile, a healthy diet pattern can not only slow down the age-related decline in physical function but also reduce the occurrence of adverse events [6]. In addition, cognitive training is an intervention measure with high operability and no obvious adverse reactions. It can promote cognitive ability and daily living ability for older adults at any stage of cognitive impairment or with normal cognitive levels [7,8]. The Integrated Care for Older People (ICOPE) guidelines, released by the World Health Organization [9], has proposed recommendations for exercise, nutrition, and cognitive interventions for older adults. Multidomain intervention by simultaneously targeting excercise, cognitive and nutritional domains has the potential to synergistically enhance functional ability and slow down age-related declines.

Several multidomain intervention studies have utilized a combination form of cognitive, exercise, and nutritional interventions. For instance, the Finnish Geriatric Intervention Study to Prevent Cognitive Impairment and Disability (FINGER) included older adults at risk of cognitive decline, indicating that multidomain intervention could reduce the occurrence of cognitive impairment and cardiovascular events [10,11]. The Frailty Intervention Trial (FIT) in Singapore developed by Tze Pin Ng et al. demonstrated a 6-month multidomain intervention significantly decreased the severity of frailty and sarcopenia in pre-frail and frail older adults [12,13]. Additionally, the Taiwan Health promotion Intervention Study for Community Elders (THISCE) revealed improvements in physical and cognitive function, as well as addressing malnutrition and depression in pre-frail and frail older adults [14]. However, the Multidomain Alzheimer Preventive Trial (MAPT) in France, which targeted older adults with subjective memory impairment, did not show a significant impact of multidomain intervention on cognitive function, muscle strength, or intrinsic capacity (IC) [15–17]. While the combination of exercise, cognitive, and nutritional interventions has been employed in studies addressing frailty, sarcopenia, depression, and cognitive impairment among common geriatric syndromes, this multidomain intervention strategy has not been explored in the field of aging with biological age as the primary outcome. Therefore, this study aims to implement cognitive, exercise, and nutritional interventions as a practical approach for multidomain intervention in the clinical practice of aging research.

The epigenetic clock is constructed from DNA methylation modifications at cytosine-phosphate-guanine (CpG) sites in the human genome. It uses mathematical algorithms to aggregate CpG sites associated with age, enabling the estimation of an individual’s epigenetic age [18]. Epigenetic age represents a biological age derived from methylation data and can predict mortality and age-related diseases such as cardiovascular diseases, Alzheimer’s disease, and cancer [19]. Given the potential reversibility of epigenetic age, an increasing number of aging studies are exploring interventions aimed at decelerating epigenetic aging [20]. Accordingly, this study will use epigenetic clocks as the primary outcome measure for aging.

The outcome measures characterizing aging are not singular. Authoritative scholars recommend the incorporation of diverse aging-related indicators as alternative aging endpoints in the same study to enhance the understanding of aging [21]. We include frailty and IC as secondary outcomes for aging in this study. IC refers to the overall status of an individual’s physical and mental abilities across five domains: locomotor, vitality (nutrition), cognitive, psychological, and sensory functions [22]. The introduction of IC signals a shift in focus within healthy aging towards keeping optimal physiological reserves in older adults [23]. Frailty and IC seem like two sides of one coin. Frailty, indicative of the accumulation of physical deficits with age, represents a negative state, while IC signifies a positive state reflecting functional reserves. IC not only complements the health information provided by frailty, but also serves as a dynamic indicator that can be monitored longitudinally throughout the lifespan [24]. Interventions targeting IC are currently in the early stages of research and are anticipated to become a focal point in the geriatric medicine [25].

In summary, this study will develop a multidomain intervention program tailored for community-dwelling older adults, integrating cognitive, exercise, and nutritional interventions. Epigenetic age accleration and pace of aging will be used as the primary outcome measure, while frailty and IC will serve as secondary outcome measures to characterize aging. Subsequently, a 6-month multidomain intervention study will be conducted to investigate the effects of the intervention on delaying aging in community-dwelling older adults.

## Methods

### Study design

The MIDA study employs a Zelen-design for randomized controlled trial [26], which combines elements of an observational study with post-randomization informed consent and a randomized controlled trial. The cognitive, exercise, and nutritional interventions in the multidomain approach require close participant cooperation under the guidance of healthcare professionals, especially in individual home-based interventions. Maintaining blinding of assigned groups to participants poses a challenge. Furthermore, traditional randomization methods may result in decreased participant compliance, increased attrition rates, and higher demands for sample size and funding. Therefore, the Zelen-design is suitable for randomized controlled trial where maintaining blinding is difficult. It allows participants to provide further informed consent based on individual circumstances post-randomization. The Zelen-design can enhance compliance with follow-up procedures, align with real-world clinical settings, and better reflect clinical practice realities.

This study employed the Proactive Health Management Platform for Older Adults (SilverHealth Pro Platform, SPP), which was developed by the Fujian Provincial Center for Geriatrics. This study protocol has been approved by the Ethics Committee of Fujian Provincial Hospital (K2023-04-039) and registered with the Chinese Clinical Trial Registry (ChiCTR2300072010). The trial protocol is in accordance with the CONSORT guidelines [27].

### Participants and recruitment

The trial will be conducted at the Fujian Provincial Center for Geriatrics. Recruitment will be conducted through printed posters in outpatient departments of Fujian Provincial Hospital and community health service centers, as well as electronic poster dissemination *via* WeChat groups and social media accounts. Interested participants will register by contacting the recruitment staff. Physicians from the Fujian Provincial Center for Geriatrics will carry out the screening according to the inclusion and exclusion criteria.

### Inclusion and exclusion criteria

Inclusion criteria: (1) aged between 60 and 85 years old; (2) living in the community; (3) a smartphone user.

Exclusion criteria: (1) severe neurological disorders (e.g. Alzheimer’s disease, vascular dementia, Parkinson’s disease, history of stroke with significant residual disability), severe psychiatric disorders (e.g. active major depressive disorder, schizophrenia or bipolar disorder), or moderate to severe cognitive dysfunction (MiniMental State Examination score ≤ 20); (2) conditions causing significant motor function impairment (e.g. severe arthritis, spinal cord injury, or muscular dystrophy), or recent fractures or surgeries within the past 6 months that limit mobility; (3) severe speech, language, hearing, or visual impairments that hinder communication with study personnel or participation in the intervention; (4) inability to perform basic activities of daily living independently, as defined by a Barthel Index score ≤ 40 or a Katz Index score ≤ 2; (5) terminal malignant diseases with a life expectancy of less than 6 months; (6) inability or unwillingness to provide informed consent or agree to follow-up.

### Calculation of sample size

Sample size was firstly established by taking reference from a intervention study with the similar primary outcome (epigenetic age acceleration) [28]. In this study, 57 subjects were randomized to intervention and 58 subjects to the control groups, with a significant reversal of 0.66 years for epigenetic age acceleration. Nevertheless, given the secondary outcome frailty might require more sample size to achieve a significant improvement (the steps in Fried criteria are quite big), we referenced a multidomain study with 6-month interventions and 12-month follow up about frailty reversal [13].

In our study, each group will have 74 subjects to have 90% power to detect significant differences at *p* < 0.05 in epigenetic age acceleration and frailty changes at the 12-month follow-up based on a mixed linear model. Moreover, we considered the 20% dropout rate and 75% adherence [17], a total of 248 individuals will be recruited with 124 individuals in each group to ensure the efficacy of the study. Participants will be matched for chronological age, gender, smoking status, BMI for each group.

### Randomization, blinding, and allocation concealment

Participants who meet the inclusion and exclusion criteria will be randomized into intervention and control groups in a 1:1 ratio. The randomization process will be carried out by a data custodian not involved in the study. The data custodian will generate a set of random numbers using a simple randomization procedure and place into sequentially numbered, sealed, opaque envelopes. The random numbers will be assigned to participants in numerical order. Participants in the intervention group will be informed about the intervention procedure and follow-up plan, while participants in the control group will be informed about the follow-up plan. If participants agree to their assigned plan, they will sign the informed consent and undergo further baseline assessment.

Due to the difficulty of blinding intervention implementers and participants in a multidomain intervention, data collection personnel and statistical analysts will be blinded to reduce bias. Participants are instructed not to disclose their group assignment to anyone during baseline and follow-up assessments.

### Control group

Participants in the control group will attend a monthly health education lecture series conducted by the Fujian Provincial Center for Geriatrics, totaling 6 sessions. The content will include scientific exercise knowledge, dementia prevention, principles of a healthy diet, management of cardiovascular risk factors, and prevention of frailty and sarcopenia in older adults.

### Intervention group

Participants in the intervention group will receive a multidomain intervention including cognitive training, exercise training, and nutritional guidance for a duration of 6 months. The form of the multidomain intervention involves a combination of group and individual interventions. The group intervention sessions will be scheduled biweekly for a total of 12 sessions. Each session will last for 120 min, consisting of 60 min cognitive training, 45 min exercise training, and 15 min nutritional guidance.

**Figure 1.**
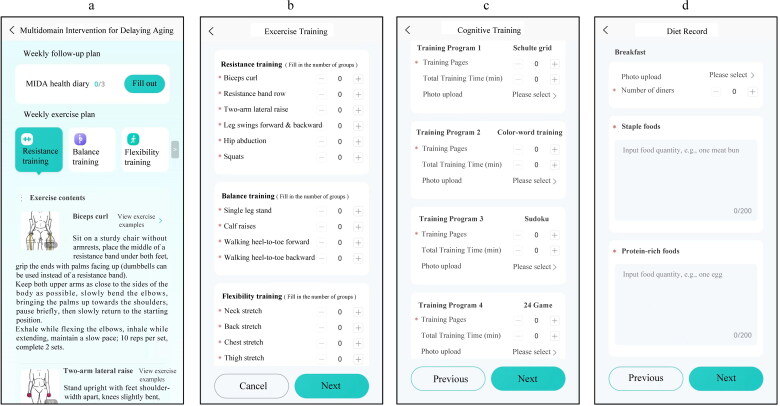
Example of mobile check-in records through the ‘SilverHealth Pro Platform’ app in the individual family intervention. a: The main interface of the app allows participants to begin documenting their exercise training, cognitive training, and dietary records by clicking on ‘MIDA Health Diary’ Participants can view exercise examples in the form of pictures or videos by selecting different exercise options. b: In the exercise training section, participants will log the number of completed groups for each exercise movement. c: In the cognitive training section, participants will record the number of pages completed or the duration spent on each cognitive task from the cognitive training workbook. If needed, they can also upload pictures of the completed tasks. d: In the dietary record section, participants will sequentially log their daily meals, including staple foods, protein-rich foods, vegetables, fruits, and fats. If needed, they can upload pictures of their meals and add notes about the number of diners. This figure originates from software owned by the Fujian Provincial Center for Geriatrics and Fujian Provincial Institute of Clinical Geriatrics.

After each group intervention session, participants will receive a physical booklet containing the content of the day’s cognitive, exercise, and nutritional intervention, and assignments for the individual family intervention (Supplementary materials). The goals of the individual family intervention include engaging in exercise training, cognitive training, and maintaining a record of healthy eating habits three times a week. Participants will log using the ‘SilverHealth Pro Platform’ app through mobile check-in records (Figure 1).

### Cognitive intervention

In the aspect of cognitive intervention, the China’s Cognitive Training Guideline indicates that cognitive training is suitable for cognitively normal older adults, those with subjective cognitive decline, mild cognitive impairment, and dementia patients. Cognitive training uses systematically designed tasks to target cognitive domains such as attention, memory, and logical reasoning, with adaptive difficulty levels to enhance individual cognitive function [29]. The recommended approach covers various cognitive domains, including but not limited to memory, attention, reasoning, and executive functions. The training dosage suggests a minimum of 30 min per session, at least three training sessions per week, with a total training duration of no less than 20 h to achieve significant effects, meaning continuous training for at least three months. Based on the framework provided by the guideline and practical content from previous studies [30–33], we outline attention training (e.g. Schulte grids, color-word training), memory training (e.g. picture memorization, recall training), logical reasoning (e.g. sudoku, matchstick training), and executive function training (e.g. finger exercises, building blocks) (Supplementary Table 1).

During each group intervention session, cognitive training will last for 60 min, covering 2 ∼ 3 training methods to ensure a balance between reinforcing existing knowledge and acquiring new training content. The primary objectives of the group intervention include enabling participants to master training methods in different cognitive domains for independent application during individual family intervention; teaching practical memory strategies to help participants better address daily challenges; and promoting participant engagement through face-to-face discussions and interactions while enhancing their sense of social involvement.

The cognitive training assignments for individual family intervention will include all the training content that has been taught. The assignments will be designed to meet the weekly training requirements of participants. Participants will record their individual family intervention training activities three times a week on the SilverHealth Pro Platform. Based on participants’ feedback on the difficulty and completion of training tasks, medical staff will make corresponding adjustments to the training content.

### Exercise intervention

According to the International Exercise Recommendations in Older Adults [5], older adults should engage in 150 min moderate-intensity aerobic exercise per week or 75 min vigorous-intensity aerobic exercise, along with muscle-strengthening exercises (resistance training) on ≥2 days. The Physical Activity Guidelines for Americans [34] recommends that the most suitable exercise regimen for older adults to maintain health is the multicomponent exercise, involving balance training, resistance training, and at least moderate-intensity aerobic exercise training ≥3 times a week, with each session lasting 30 ∼ 45 min. Therefore, we will adopt a multicomponent exercise training approach, including aerobic exercise, resistance training, balance, and flexibility training. The training objective is to combine group intervention with individual family intervention ≥3 times a week, with each session lasting ≥45 ∼ 60 min.

The group intervention for exercise training will last 45 min each time, incorporating resistance training, balance training, and flexibility training (Supplementary Table 2). As the group intervention progresses, we gradually introduce the use of resistance bands, dumbbells, and sandbags to increase difficulty in resistance movements, aiming for progressive resistance training. The group intervention helps participants grasp the key points of multicomponent exercises and provides training charts and videos for individual family interventions. Additionally, we will provide participants with a recommended list of aerobic exercises, allowing participants to autonomously choose suitable types of aerobic exercises for individual family interventions. Based on the participants’ completion of exercises during the group intervention and feedback on exercise training during individual family interventions, medical staff will provide personalized exercise recommendations.

We will require participants to exercise with support (such as a chair or railing) to ensure the safety of the exercise process. Intermittent rest breaks and hydration will be provided throughout the exercise session. Medical staff will inform participants that they may experience delayed onset muscle soreness as a normal response to exercise due to muscle adaptation. If participants feel pain or discomfort or are unable to tolerate the exercise, they will be advised to rest or adjust the intensity of the exercise.

### Nutritional intervention

Nutritional intervention consists of dietary guidance, nutritious meal demonstration, and nutrition lectures. Nutritional guidance has the advantages of being effective in intervention and highly operational, helping older adults to change their daily diets and establish healthy eating habits [35], thereby enhancing their nutritional literacy and improving their nutritional status [36].

Nutritionists will apply the Dietary Guidelines for Chinese [37] as a guiding basis and emphasize the importance of a balanced diet for older adults during the nutritional guidance process. The group intervention for nutritional guidance will last 15 min each session. The first two sessions will primarily introduce the dietary structure and guidelines for older populations, providing quantified charts of common foods to help participants understand the types and portions of food they should consume daily. The following eight sessions will be conducted in the form of themed lectures to guide older adults on healthy eating habits, with three nutrition meal demonstrations provided to assist participants in developing suitable dietary plans (Supplementary Table 3). Furthermore, common dietary questions from participants will be addressed one by one during every group intervention session.

Participants will be required to complete dietary logs three times a week for individual family interventions, recording the staple foods, protein sources, vegetables, fruits, lipids, etc., consumed during breakfast, lunch, and dinner in the form of pictures or text. Medical staff will evaluate the adequacy of the participants’ diets based on their individual nutritional status, provide feedback for improvement, and respond through the SilverHealth Pro Platform.

### Outcome measures

**Figure 2.**
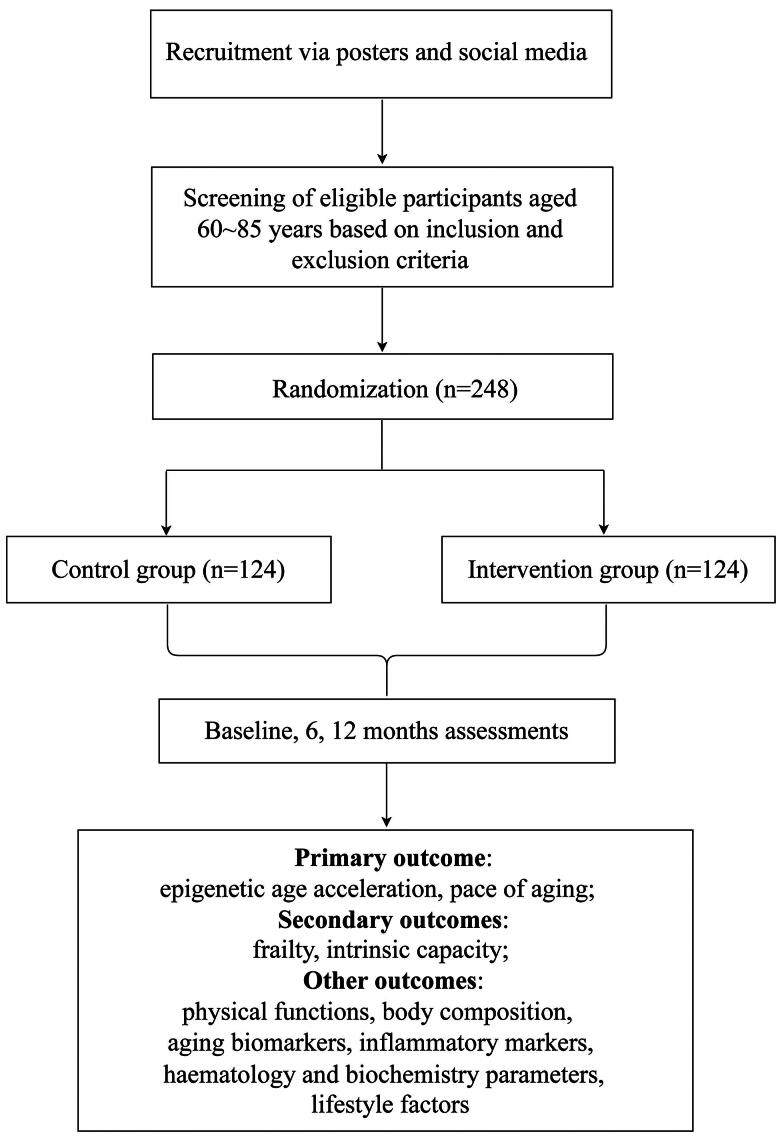
Flow chart of multidomain intervention.

Trained data collectors will conduct questionnaires and physical examinations with the participants. Additionally, the participants will undergo blood tests and body composition analysis. Data will be collected at three different times: post-randomization baseline, 6-month, and 12-month timepoints. The flowchart of the study protocol is displayed in Figure 2. Data collectors and data entry personnel neither participate in the intervention nor are aware of the participants’ group assignments.

The primary outcomes are the change in epigenetic age acceleration and pace of aging. We will apply eight representative aging clocks in our primary outcomes: Horvath, Hannum, GrimAge, PhenoAge, DunedinPACE, GrimAge version 2, PC-PhenoAge, and PC-GrimAge. Epigenetic age acceleration is defined as the residuals of the linear regression of DNA methylation age on chronological age, which can be calculated by seven clocks (Horvath, Hannum, GrimAge, PhenoAge, GrimAge version 2, PC-PhenoAge, and PC-GrimAge). The rate of aging represented by DunedinPACE is also the primary outcome we are concerned about.

The secondary outcomes are the changes in frailty score and IC Z-score. Other outcomes include physical functions, body composition, aging biomarkers, inflammatory markers, haematology and biochemistry parameters, and lifestyle factors (Table 1).

**Table 1.** Overview of outcomes and assessment time points.

Outcomes	Outcome measures	Screening	Baseline	6 months	12 months
Primary outcomes					
Epigenetic age acceleration and pace of aging	Horvath, Hannum, GrimAge, PhenoAge, DunedinPACE, GrimAge version 2, PC-PhenoAge, and PC-GrimAge		√	√	√
Secondary outcomes					
Frailty	Fried phenotype	√	√	√	√
Intrinsic capacity	IC Z-score: cognition: MMSE; locomotion: SPPB; vitality: MNA; psychology: GDS-15	√	√	√	√
Other outcomes					
Physical functions	Handgrip strength	√	√	√	√
	Gait speed: 4-m walk test	√	√	√	√
	Timed up-and-go test	√	√	√	√
	Five-times sit-to-stand test	√	√	√	√
Body composition	BMI, waist-to-hip ratio, skeletal muscle mass, skeletal muscle index, body fat percentage, visceral fat area, phase angle	√	√	√	√
Aging biomarkers	Telomere length		√	√	√
	Mitochondrial DNA copy number		√	√	√
Inflammatory markers	hsCRP, homocysteine		√	√	√
	IL-1β, IL-2, IL-4, IL-6, IL-8, IL-10, IL-13, IFN-γ, TNF-α, MCP-1, Eotaxin, CXCL9, CXCL10, CX3CL1, sTNF-R1, sTNF-R2, G-CSF、GM-CSF, VEGF, RANTES, TRAIL		√	√	√
Haematology and biochemistry parameters	Hemoglobin, platelets, neutrophils, hematocrit, white blood cell count; blood urea nitrogen, creatinine, uric acid, glucose, albumin, total bilirubin, alkaline phosphatase, alanine transaminase (ALT), aspartate transaminase (AST), lipid profile		√	√	√
Lifestyle factors	Dietary diversity	√	√	√	√
	Physical activity and sedentary time	√	√	√	√
	Health literacy	√	√	√	√

IC: intrinsic capacity; MMSE: Mini-Mental State Examination; SPPB: Short Physical Performance Battery; MNA: Mini Nutritional Assessment; GDS-15: Geriatric Depression Scale-15; BMI: body mass index; hsCRP: high-sensitivity C-reactive protein. IL-1β: Interleukin-1 beta; IL-2: Interleukin-2; IL-4: Interleukin-4; IL-6: Interleukin-6; IL-8: Interleukin-8; IL-10: Interleukin-10; IL-13: Interleukin-13; IFN-γ: Interferon-gamma; TNF-α: Tumor Necrosis Factor-alpha; MCP-1: Monocyte Chemoattractant Protein-1; CXCL9: C-X-C Motif Chemokine Ligand 9; CXCL10: C-X-C Motif Chemokine Ligand 10; CX3CL1: C-X3-C Motif Chemokine Ligand 1; sTNF-R1: Soluble Tumor Necrosis Factor Receptor 1; sTNF-R2: Soluble Tumor Necrosis Factor Receptor 2; G-CSF: Granulocyte Colony-Stimulating Factor; GM-CSF: Granulocyte-Macrophage Colony-Stimulating Factor; VEGF: Vascular Endothelial Growth Factor; RANTES: Regulated on Activation, Normal T Cell Expressed and Secreted; TRAIL: Tumor Necrosis Factor-Related Apoptosis-Inducing Ligand.

### Epigenetic age acceleration and pace of aging

Our study will employ eight representative epigenetic aging clocks to estimate epigenetic age acceleration and pace of aging: Horvath [38], Hannum [39], PhenoAge [40], GrimAge [41], DunedinPACE [42], GrimAge version 2 [43], PC-PhenoAge and PC-GrimAge [44].

The first-generation epigenetic clocks, the Horvath clock and the Hannum clock, are constructed solely based on DNA methylation data. Horvath [38] developed an epigenetic clock comprising 353 CpG sites by analyzing publicly available DNA methylation datasets from a wide range of human tissues and cell types. Hannum et al. [39] created an epigenetic clock based on 71 CpG sites, utilizing DNA methylation data from the blood of adults. Both the Horvath and Hannum clocks exhibit high accuracy in predicting chronological age.

The second-generation epigenetic clocks, such as GrimAge and PhenoAge, incorporate clinical biomarkers in addition to methylation data. The PhenoAge clock integrates 9 clinical blood biomarkers (albumin, alkaline phosphatase, creatinine, C-reactive protein, glucose, mean corpuscular volume, red cell distribution width, white blood cell count, and lymphocyte percentage) and 513 age-related CpGs [40]. The GrimAge clock [41] is composed of 7 DNA methylation-based plasma protein biomarkers, pack-years of smoking, and 1030 CpG sites. The plasma protein biomarkers include cystatin C, leptin, tissue inhibitor of metalloproteinase 1 (TIMP-1), adrenomedullin (ADM), beta-2-microglobulin (β2-M), growth differentiation factor 15 (GDF-15), and plasminogen activation inhibitor 1 (PAI-1) [41]. Both GrimAge and PhenoAge demonstrate strong correlations with lifestyle risk factors and perform well in predicting morbidity and mortality risks.

The third-generation epigenetic clocks, such as DunedinPACE [42], GrimAge version 2 [43], PC-PhenoAge and PC-GrimAge [44], are characterized by their integration of multi-dimensional data, optimization of algorithms and models, and ability to comprehensively assess individual health status, disease risk, and the effects of aging interventions. DunedinPACE [42] is developed based on longitudinal changes in multi-system biomarkers, reflecting the pace of aging, with its unit expressed as ‘years/calendar year’. GrimAge version 2 [43] expandes upon the original GrimAge by incorporating additional DNA methylation (DNAm)-based surrogates for clinically relevant plasma proteins (DNAm logCRP and DNAm logA1C). PC-PhenoAge and PC-GrimAge [44] are epigenetic clocks optimized based on principal component analysis (PCA). They utilize principal components derived from the CpG sites of the foundational PhenoAge and GrimAge clocks, offering high reliability and potential applications in personalized medicine, longitudinal tracking, *in vitro* studies, and clinical trials of aging interventions.

We have introduced a novel variable, ‘Mean EpiAge’ [45], which is calculated as the average methylation age derived from seven established epigenetic clocks: Horvath, Hannum, GrimAge, PhenoAge, GrimAge version 2, PC-PhenoAge, and PC-GrimAge. Notably, DunedinPACE is excluded from the computation of Mean EpiAge because it specifically quantifies the pace of aging rather than providing a methylation age estimate. The development of Mean EpiAge aims to reconcile discrepancies between DNA methylation age and chronological age that arise from the use of different epigenetic clocks, thereby offering a more comprehensive measure of biological aging. We will focus on the results of each epigenetic clock because they enable us to conduct comparisons with studies that utilize the same clocks. Similarly, we pay attention to the Mean EpiAge, as it represents an overall and comprehensive aging outcome.

Genomic DNA from blood samples will be extracted using the QIAamp 96 DNA QIAcube HT Kit and processed on the QIAcube HT automated nucleic acid extractor. Then we will employ the 935K DNA methylation array (Infinium Methylation EPIC BeadChip Kit) to assess the methylation status of the whole genome. To ensure data reliability, the methylation data will undergo comprehensive processing and quality control, which includes filtering out low-quality probes, normalizing the raw methylation data (converting it to β-values within the range of 0 to 1), and conducting batch effect analysis and correction. The online tool ‘DNA Methylation Age Calculator’ (https://dnamage.clockfoundation.org) developed by Horvath et al. [38] and open-source R packages will be used to calculate DNA methylation age. A file containing the methylation β-values for all samples and a file with brief annotation information need to be uploaded. After a period of online analysis, the platform will generate output files with the calculated DNA methylation age and other estimates.

### Frailty

This study will use the Fried phenotype [46] for frailty assessment, which includes weight loss, decreased gait speed, reduced handgrip strength, decreased physical activity, and fatigue. Individuals meeting three or more criteria will be classified as frailty, those meeting one or two criteria are classified as pre-frailty, and those meeting none of the criteria are classified as robust older adults. The frailty score ranges from 0 to 5.

### Intrinsic capacity

The assessment of IC will be conducted according to the five domains proposed by the World Health Organization (WHO), including cognitive, locomotor, vitality, psychological, and sensory domains [47]. We will employ the Mini-Mental State Examination (MMSE) [48], Short Physical Performance Battery (SPPB) [49], Mini Nutritional Assessment (MNA) [50], Geriatric Depression Scale-15 (GDS-15) [51], and self-reported vision and hearing status for the evaluations of five domains.

We consider the IC by integrating the values obtained from five domains. To obtain a summary score that represents IC, we calculated Z-score. A Z-score reflects the distance of an individual’s data point from the mean of the population we studied, measured in standard deviations. The interpretation of Z-score is as follows: a positive Z-score means that an individual’s raw score is higher than the population mean, while a negative Z-score indicates that the individual’s raw score is lower than the mean. In other words, the Z-score shows the number of standard deviations by which an individual’s raw score deviates from the population mean [52].

In previous studies focusing on IC, the IC Z-score is extensively employed as a robust and well-established measurement tool for assessing IC [53]. In this study, we first calculated the Z-score for each of the five domains. Then, we computed a composite IC Z-score. This composite score was calculated by taking the sum of the Z-scores of the five domains and dividing it by five. This method standardizes the five domains to the same scale before calculating the overall summary measure. The specific calculation steps are as follows:

1. Single-domain Z-score: For each domain, the Z-score is calculated using the formula:
$$\text{Single}-\text{domain Z‐}\text{score}=\frac{\text{Individual}\prime \text{s measurement value }-\text{ Population mean value}}{\text{Population standard deviation}}$$
2. IC Z-score: The overall intrinsic capacity Z-score is obtained by averaging the Z-scores across the five domains:
$$\text{IC Z‐}\text{score}=\frac{\text{Sum of Z}-\text{scores across the five domains}}{5}$$

### Questionnaires

All assessment questionnaires will be completed by trained professional investigators through face-to-face interviews with the participants. The questionnaires will include demographic information (name, gender, age, marital status, education level, etc.), lifestyle factors (drinking, smoking, dietary diversity, physical activity and sedentary time, health literacy, etc.), medical history (hypertension, diabetes, hyperlipidemia, coronary heart disease, stroke history, etc.), and the standardized scales in comprehensive geriatric assessment.

MMSE: It assesses cognitive function, including orientation, immediate and delayed memory, attention, calculation, naming, repetition, reading, writing, and copying abilities, with a total score ranging from 0 to 30 [48].

SPPB: It evaluates balance, gait speed, lower limb strength, and functional capacity, with a total score ranging from 0 to 12 [49].

MNA: It includes evaluations of body weight, dietary intake, overall nutritional status, and measurements of mid-upper arm circumference and calf circumference, with a total score ranging from 0 to 30 [50].

GDS-15: It assesses the psychological state of older adults over the past week, focusing on mood, interest, fatigue, and feelings of self-blame through 15 questions, with a total score ranging from 0 to 15 [51].

Vision and hearing: participants select the most appropriate response to describe their condition: ①normal (0 points); ② mild decline (1 point); ③ limited in daily life (2 points); or ④ severe decline (3 points). The sensory score was calculated as the sum of the vision and hearing scores.

### Physical examinations

Handgrip strength: Handgrip strength will be measured using a electronic handheld dynamometer (Jamar, USA) while the participant is seated. Participants will apply force gradually until reaching the maximum value. Three measurements will be taken for each hand, and the highest value will be recorded [54].

Gait speed: Gait speed will be assessed by a 4-m walk test. Participants is instructed to walk a straight 4-m distance at a normal pace from the same starting point. The average time of the two trials will be taken as gait speed [55].

Timed up-and-go test: Participants will sit in a chair with armrest. After standing up, they will walk forward 3 meters at their normal walking speed. Upon crossing a marked line, they will turn around and walk back to the chair, then sit down again with their back against the chair’s backrest. The tester will record the time from when the participant’s back leaves the chair’s backrest until they are seated and leaning back against it again [56].

Five-times sit-to-stand test: Participants will sit in a chair without armrests with their hands crossed over their chest. Upon hearing the instruction, they will quickly complete five stands and sits. The tester will record the time from the start until the participant’s last sit-down [57].

### Blood tests

The nurse will take peripheral venous blood from the participant’s forearm after fasting for more than 10 h in the morning. The blood samples will be sent out for measurements of haematology and biochemistry markers, high-sensitivity C-reactive protein (hsCRP), and homocysteine levels. Additionally, 5 mL of blood will be centrifuged at 2500 rpm for 15 min at 4 °C to obtain serum and plasma, then stored in the −80 °C freezer for later analysis. The Luminex-MultiDTX technology will be used to detect inflammatory markers in plasma. Telomere length and Mitochondrial DNA copy number will be measured using DNA samples with quantitative real-time polymerase chain reaction (q-PCR). Each reaction will be performed in triplicate and expressed as 2(-ΔΔCt).

### Body composition

This study will use the InBody 770 (Biospace, Korea), a body composition analyzer based on bioelectrical impedance analysis (BIA), to measure body composition. Participants are required to remove their coats and shoes, take off any metal accessories, and wear light clothing during the measurement. They will stand on the platform, grasping the hand electrodes with both hands and ensuring that their heels and the balls of their feet are properly placed on the foot electrodes. The body composition indicators used in this study will include body mass index (BMI), waist-to-hip ratio, skeletal muscle mass, skeletal muscle index, body fat percentage, visceral fat area, and phase angle.

### Statistical analysis

This study will follow the intention-to-treat principle for statistical analysis, including participants who complete baseline assessments and at least one follow-up assessment in the final analysis. Normally distributed continuous variables with a normal distribution will be presented as mean ± standard deviation, while non-normally distributed continuous variables will be presented as median (interquartile range). Categorical variables will be presented as frequencies and percentages (%). For the baseline data comparison between the control group and the intervention group, independent samples t-test will be used for normally distributed continuous variables, Mann-Whitney U test for non-normally distributed continuous variables, and chi-square test or Fisher’s exact test for categorical variables. Repeated measures analysis of variance will be used to analyze the changes in primary and secondary outcomes within the intervention and control groups, with multiple comparisons corrected using Bonferroni adjustment. Repeated measures analysis of covariance will be employed to compare the differences in primary and secondary outcome measures between the intervention and control groups at different follow-up times. The statistical analysis will be conducted using R language version 4.0.3 and SPSS 25.0 statistical software. Hypothesis testing will be conducted using two-tailed tests with a significance level of α = 0.05, where *p* < 0.05 is considered statistically significant.

### Safety assessments

In our multidomain intervention strategy, the exercises are submaximal, and the primary form of nutritional intervention is dietary guidance. Therefore, the multidomain intervention in this study is considered safe, and no adverse events are expected during the intervention period. Nonetheless, any adverse events will be documented and reported to the principal investigators and the ethics committee within 24 h.

## Discussion

In the field of geriatrics, the exploration of interventions to delay aging is an emerging topic that promotes healthy aging. From an economic perspective, extending healthy life expectancy by 2.2 years could save $7 trillion in social healthcare expenditures over 50 years [58]. Exploring interventions to delay aging is considered a more meaningful medical investment than researching disease treatments. It not only helps extend the healthy lifespan of older adults but also brings significant benefits to public health and healthcare systems [3]. Therefore, we believe that multidomain intervention can be applied in the field of aging. It aligns with the goal of healthy aging, has the potential to maintain physical function and combat aging, and serves as proactive health-promoting measures for older populations.

This study strictly references the research designs of completed or ongoing multidomain intervention studies internationally. For example, FINGER [59] aims to investigate the efficacy of multidomain intervention in preventing cognitive impairment, involving populations from six regions in Finland. The two-year intervention duration covers dietary adjustments, physical exercise, cognitive training, and cardiovascular risk management. Xu et.al [33] implemented the SINGER (SINgapore GERiatric Intervention Study to Reduce Cognitive Decline and Physical Frailty) study aimed at slowing cognitive decline and frailty. In Malaysia, the WE-RISE study [32] and the AGELESS study [60] have been conducted to reverse cognitive decline. Chen LK et.al [14] also carried out the THISCE study to improve physical frailty and cognitive function among community-dwelling older adults.

We drew inspiration from the above-mentioned studies in our intervention model, combining group interventions with individual family-based interventions. The group intervention involves a physician-led 2-hour session where older adults will participate in cognitive training 60 min, exercise training 45 min, and nutritional intervention 15 min. The duration of the group intervention is designed considering the limited energy and attention span of older adults. The group intervention will be held biweekly for a total of 10 sessions, differing from the gradual reduction in intervention frequency seen in other studies. For example, the THISCE study spanned 12 months with weekly sessions in the first month, biweekly sessions in the second month, and monthly sessions for the remaining 10 months [14]. Given that our study is a short-term intervention lasting 6 months, to ensure participants’ training volume and compliance, we consider that a biweekly group intervention frequency may be suitable in practice.

Currently, some scholars have conducted intervention studies targeting biological age. Most of these studies have implemented dietary and exercise interventions in obese populations. For example, a one-year intervention combining diet and exercise in 107 obese older adults found that both the diet group and the diet-plus-exercise group showed a reduction in Klemera Doubal Method (KDM) biological age at the 6-month and 12-month follow-up assessments. Specifically, at 6 months, the diet group showed a reduction of 1.4 years, while the diet-plus-exercise group showed a reduction of 1.7 years. At 12 months, the diet group showed a reduction of 2.4 years, and the diet-plus-exercise group showed a reduction of 2.2 years [61]. In the Israeli CENTRAL study, 120 obese individuals underwent an 18-month weight-loss intervention combining diet and exercise, which resulted in a 7.1-month delay in methylation age compared to the expected aging in the older male subgroup [62]. Similarly, a U.S. study involving 16 obese older adults implemented a 3-month intervention consisting of dietary counseling and a combination of aerobic and resistance exercise. However, this study found no statistically significant reduction in three types of methylation age (Horvath, Hannum, and PhenoAge clocks) after the intervention [63]. In the widely recognized CALERIE study on caloric restriction, 128 normal-weight or mildly overweight participants underwent a 25% caloric restriction for 2 years. The pace of aging, as measured by DunedinPACE, slowed by 2%∼3% in the caloric restriction group (equivalent to a 10%∼15% reduction in mortality risk), but no significant changes were detected using the other two aging clocks (PC-PhenoAge and PC-GrimAge) [64].

Additionally, some studies have explored aging interventions in relatively healthy populations. The European NU-AGE study [65] enrolled 120 older adults aged ≥65 years (60 Italians and 60 Poles) to assess the effects of a 1-year Mediterranean diet intervention on methylation age. The results showed that only the Polish female subgroup experienced a reduction in Horvath methylation age acceleration by 1.47 years, suggesting that the Mediterranean diet may promote epigenetic rejuvenation, although this effect varied by country, gender, and individual specificity. Fitzgerald et al. [66] first conducted a 2-month multi-domain intervention involving diet, exercise, sleep, and relaxation guidance in 43 healthy adult males aged 50–72 years, which resulted in a reduction of Horvath methylation age by 3.23 years. Subsequently, they tested the same intervention in 6 females aged 46–65 years, and the participants’ methylation age decreased by an average of 4.6 years after the intervention [67].

Looking at the above intervention studies using methylation age or KDM biological age as outcome measures, most studies were not originally designed with aging as the primary focus, leading to some bias in the study populations. Additionally, variations in sample size, ethnicity, gender, and specific outcome measures limit the generalizability of these findings. Nevertheless, the diverse intervention strategies explored in these studies offer valuable insights for researchers. While the majority of prior studies have focused on combined diet and exercise interventions, the multidomain intervention in this study integrates cognitive training, exercise training, and nutritional guidance. To date, limited literature has applied a similar intervention protocol to methylation age. Our study may provide new evidence for clinical interventions targeting aging. Furthermore, the aforementioned studies on the reversal effects of methylation age involved intervention durations ranging from 2 months to 2 years. Considering that our intervention encompasses a broader range of components, we have established a moderate intervention duration of 6 months. We hypothesize that this multi-domain intervention may have a decelerating effect on aging.

In the design of individual family-based intervention programs, we have integrated the use of traditional paper materials and mobile medical platforms. Participants can refer not only to paper exercise charts but also follow exercise training videos when exercising at home. The mobile medical platform can assist healthcare providers in monitoring and supervising the participants’ home training progress, while also providing opportunities for communication and feedback between participants and healthcare providers, thus facilitating the entire intervention process. The older population is likely to encounter some challenges in learning to use the mobile medical platform, but this also presents an opportunity for them to enhance their daily living and cognitive abilities, as well as integrate into society. In our group interventions, we will provide operational training on platform usage for older participants and encourage their family members to also participate in guiding home interventions. The combined form of group interventions with individual family-based interventions aims to enable participants to apply what they learn in group interventions to their daily lives, gradually cultivating healthy lifestyle habits. Our intervention approach emphasizes that participants should not solely rely on healthcare providers for completing the intervention but rather prioritize their own proactive engagement in training at home. This approach aligns with the concept of active health promotion among older adults.

Our study may provide compelling evidence that practical and sustainable multidomain intervention measures could be implemented using simple materials and facilities. The auxiliary equipment used in exercise training such as resistance bands, dumbbells, and sandbags are readily available and can be used at home, even with all exercises achievable using just a resistance band. Materials for cognitive training and nutritional guidance include booklets, visual aids, or cards, all of which are low-cost and reusable. While the primary goal of this study is not to assess the cost-effectiveness of multidomain intervention, our design considers economic feasibility, with intervention costs being relatively affordable, independent of specialized medical environments or equipment, thus saving healthcare resources.

In this study, we chose epigenetic clocks as the primary outcome measures due to its well-established role as a biomarker of biological aging and its sensitivity to lifestyle interventions. Geriatricians can utilize the DNA methylation age to explore the underlying mechanisms of aging, investigate factors related to aging, and evaluate the effectiveness of aging interventions [68]. In addition, there are dedicated online platforms and open-source R packages for calculating the methylation age. This enables the use of methylation age as a biomarker for quantifying aging in different populations, and also makes the results of various studies comparable and generalizable. If this study is successfully implemented, it will provide valuable experiences and models for health management and aging intervention for older populations in other regions. With the context of an aging society, our findings may provide new insights for public health managers in promoting proactive health, which involves leveraging existing healthcare resources and community networks to enhance the health and well-being of older adults.

There are also inherent limitations to the study design. Participants and the intervention staff could not be blinded due to the nature of our intervention contents. We do not consider this to be a significant issue as we will employ blinding for outcome assessors and utilize a Zelen-design for randomized controlled trial which is more in line with clinical practice reality. Additionally, we plan to conduct one-to-many teaching operations in the group intervention rather than one-to-one due to manpower costs. While this may impact the intervention’s effectiveness, it is a common issue when generalizing intervention models to more community healthcare institutions with more participants. We need to explore the most cost-effective intervention strategy in future practice. Moreover, our study will exclude participants with moderate to severe cognitive dysfunction (MMSE score *≤* 20) to ensure ethical rigor and protocol compliance. Although this approach enhances the internal validity of our findings, it may limit the generalizability of the IC results to populations with cognitive impairment. Future studies should explore IC in individuals with cognitive dysfunction to provide a more comprehensive understanding of this construct.

Another limitation of the current study is that we evaluated the combined effect of cognitive training, exercise, and nutrition interventions without isolating the individual contributions of each component. This approach was adopted due to practical considerations, including sample size limitations and the complexity of the study design. In future studies, we plan to first conduct a single-center pilot intervention study, followed by a multi-center study with a larger sample size. In the multi-center study, participants will be randomized to different intervention groups (e.g. cognitive training alone, exercise alone, nutrition alone, and their combinations) and followed up for a longer period of time. This design will allow for a more precise evaluation of the independent and interactive effects of each intervention component.

Although the SilverHealth Pro Platform employed in this study presents several advantages, like real-time data collection and personalized feedback, it may pose usability challenges for older adults. Potential barriers include limited familiarity with technology and psychological resistance to new tools. To address these challenges, we are implementing some improvement strategies, such as simplifying the user interface, incorporating voice-assisted features, providing hands-on training, and offering ongoing technical support. In the future, we plan to recruit a small group of older adults to test the SilverHealth Pro Platform. Based on the participants’ feedback, we will optimize the platform’s design to enhance its usability and improve user compliance. In addition, we will provide alternative solutions (such as paper-based records or telephone follow-ups) for older individuals who indeed have difficulty using the mobile health platform.

To the best of our knowledge, there is currently no literature reporting on the multidomain intervention involving cognition, exercise and nutrition for delaying aging. Therefore, the present study aims to introduce a new approach to delaying aging and offer a practical multidomain intervention strategy for healthcare institutions.

## Supplementary Material

Supplemental Material

## Data Availability

The data that support the findings of this study are available from the corresponding author upon reasonable request through e-mail (Pengli Zhu: zpl7755@hotmail.com).
