# Supplementary material for: Multidomain intervention for delaying aging in community-dwelling older adults (MIDA): study design and protocol
Source: Ann Med. 2025 Apr 29;57(1):2496409. doi: 10.1080/07853890.2025.2496409 (PMC12042233; doi:10.1080/07853890.2025.2496409)
Supplement: Supplemental Material [file IANN_A_2496409_SM4210.zip › Suppl_Data/IANN-2024-5738.R1-supplementary_materials_Clean copy.docx]

Supplementary materials

**Article title: Multidomain Intervention for Delaying Aging in Community-dwelling Older Adults (MIDA): Study Design and Protocol**

**Supplementary Table 1** Scheme of cognitive training

**Supplementary Table 2** Scheme of exercise training

**Supplementary Table 3** Scheme of nutrition guidance

In the cognitive training, our study primarily utilizes traditional methods of pen-and-paper exercises and hands-on operations without exploring new approaches based on virtual reality or artificial intelligence technologies. Although computer-assisted cognitive training has been recognized for improving cognitive functions [1,2], traditional pen-and-paper training methods have also proven effective [3]. Moreover, a limitation in our cognitive training is the inability to provide personalized training programs for each participant. Despite efforts to balance the difficulty levels of tasks in each homework assignment to accommodate participants with varying cognitive levels, individualized training plans could not be tailored for each participant.

Regarding exercise training, virtually all multidomain intervention studies apply multicomponent exercise, which helps older adults improve physical functions such as muscle strength, gait speed, and balance ability [4]. Multicomponent exercise is also recommended in China’s consensus on frailty prevention and interventions for physical function impairment in older adults. Widely used international examples of multicomponent exercise include the VIVIFRAIL program [5] in Spain for frailty and cognitive decline prevention and the Otago program [6] in New Zealand for fall prevention. Our research team has accumulated a certain foundation in multicomponent exercise intervention for older adults and has published exercise charts in Chinese and articles [7,8]. We select the representative 16 movements from the charts for teaching, including 6 resistance training movements, 4 balance training movements, and 6 flexibility training movements.

During the group interventions for nutrition, we will first introduce the balanced diet guidelines for older adults, supplemented by explanations using food quantification charts, followed by demonstrations of three nutritious meals. We instruct older adults on healthy eating habits by combining theory with practical guidance, and focus on the daily intake of staple foods, proteins, fruits and vegetables, and fats. Participants engage in self-diet management during personal home interventions based on the dietary objectives mentioned above. In the subsequent nutrition lectures, we will conduct popular science education on common dietary issues and nutritional needs specific to older adults. Our nutritional intervention does not involve oral nutritional supplements because nutritional guidance serves as the foundation and primary means of intervention. Even without the addition of nutritional supplements, nutritional guidance alone can improve the nutritional status of older adults and help them enhance their self-efficacy, recognizing the importance of self-diet management [9,10].

**References**

[1] Li R, Geng J, Yang R, et al. Effectiveness of Computerized Cognitive Training in Delaying Cognitive Function Decline in People With Mild Cognitive Impairment: Systematic Review and Meta-analysis. J Med Internet Res. 2022;24(10):e38624.

[2] Chan A, Ip R, Tran J, et al. Computerized cognitive training for memory functions in mild cognitive impairment or dementia: a systematic review and meta-analysis. NPJ Digit Med. 2024;7(1):1.

[3] Bahar-Fuchs A, Martyr A, Goh AM, et al. Cognitive training for people with mild to moderate dementia. Cochrane Database Syst Rev. 2019;3(3):CD013069.

[4] Izquierdo M, Merchant RA, Morley JE, et al. International Exercise Recommendations in Older Adults (ICFSR): Expert Consensus Guidelines. J Nutr Health Aging. 2021;25(7):824-853.

[5] Casas-Herrero A, Anton-Rodrigo I, Zambom-Ferraresi F, et al. Effect of a multicomponent exercise programme (VIVIFRAIL) on functional capacity in frail community elders with cognitive decline: study protocol for a randomized multicentre control trial. Trials. 2019;20(1):362.

[6] Shubert TE, Goto LS, Smith ML, et al. The Otago Exercise Program: Innovative Delivery Models to Maximize Sustained Outcomes for High Risk, Homebound Older Adults. Front Public Health. 2017;554.

[7] Li N, Huang F, Wang N, et al. Effectiveness of a mHealth platform-based lifestyle integrated multicomponent exercise (PF-Life) program to reverse pre-frailty in community-dwelling older adults: a randomized controlled trial study protocol. Front Public Health. 2024;121389297.

[8] Li Q, Huang F, Lin W, et al. Impact of 12-Month Late-in-Life Exercise Training on Cardiopulmonary Reserve, Static Cardiac Structure, and Function: A Randomized Clinical Trial. J Am Med Dir Assoc. 2024;25(9):105117.

[9] Rea J, Walters K, Avgerinou C. How effective is nutrition education aiming to prevent or treat malnutrition in community-dwelling older adults? A systematic review. Eur Geriatr Med. 2019;10(3):339-358.

[10] Juckett LA, Lee K, Bunger AC, et al. Implementing Nutrition Education Programs in Congregate Dining Service Settings: A Scoping Review. Gerontologist. 2022;62(2):e82-e96.

**Supplementary Table 1** Scheme of cognitive training

| Domain of cognition | Specific contents | Examples |
| --- | --- | --- |
| **Attention** | **Schulte grid:** Participants are instructed to verbally identify numbers 1 to 25 while pointing to them as quickly as possible in a Schulte grid filled with randomized numbers 1 to 25, with the time taken being recorded.  Difficulty levels: 4×4 grid →5×5 grid →6×6 grid | 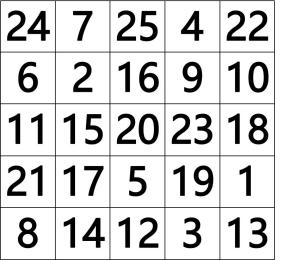 |
|  | **Color-word training:** Participants undergo color-word training in three modes in a grid filled with Chinese characters of different colors. Mode 1: Reading the characters; Mode 2: Reading the colors; Mode 3: Reading the characters first and then the colors (e.g., Mode 1: Red character; Mode 2: Yellow color; Mode 3: Red character, yellow color).  Difficulty levels: 3×3 grid →4×4 grid →5×5 grid | 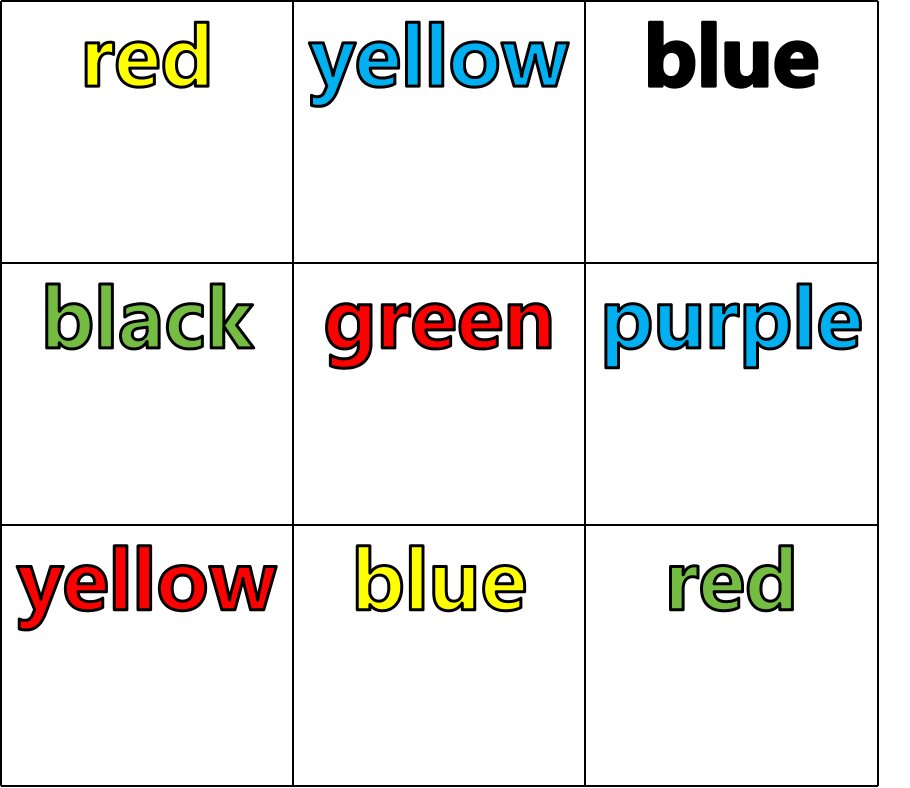 |
|  | **Spot the differences:** Participants are required to circle the differences between two images within a set time limit using a marker pen.  Difficulty levels: 4 differences →5 differences →6 differences | 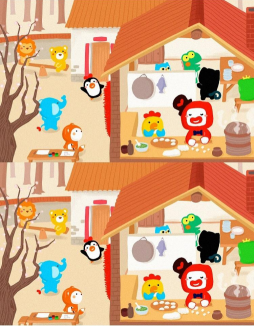 |
| **Memory** | **Playing card shorthand:** Participants are required to memorize the numbers and suits of four playing cards within a set time limit, then quickly cover the cards and write down the answers in sequence. (e.g., Spades 9, Hearts 3, Clubs 4, Diamonds 6).  Difficulty levels: Increase the number of cards dealt gradually based on training progress. | 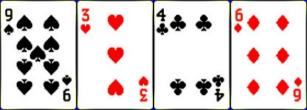 |
|  | **Card memorization:** Participants are required to memorize all items on a card within a set time limit and write them down on paper.  Difficulty levels: Reduce the memorization time or recall the card content in the form of a drawing based on training progress. | 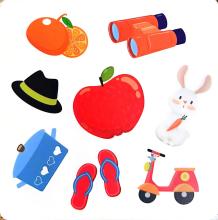 |

**Supplementary Table 1** Scheme of cognitive training (continued)

| Domain of cognition | Specific contents | Examples |
| --- | --- | --- |
| **Memory** | **Picture memorization:** Participants are required to memorize as many details as possible from one image within a set time limit, followed by recalling and answering questions about the image.  Difficulty levels: Recall the image content in written form or through drawing based on training progress. | 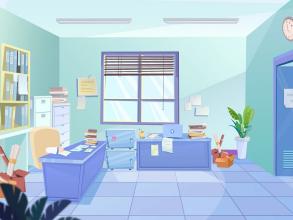 |
|  | **Recall Training:** Participants exercise their long-term memory by recalling past stories, visualizing rich details in their minds.  For example, browsing through a photo album and trying to remember the background of each old photo, then writing down things related to the photos; reminiscing about specific events from the past with family or friends, such as trips, birthdays, weddings, etc.; recalling events from the previous week each week, or reviewing the day's schedule each day; recalling the content of the last activity before each group cognitive training intervention session. | 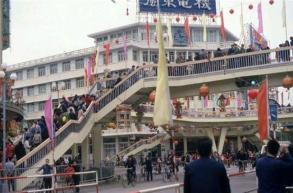 |
| **Logical reasoning** | **Sudoku:** Participants fill in numbers 1 to 6 in the blank squares, ensuring that each row, column, and sub-grid of the six grids contains unique numbers from 1 to 6.  Difficulty levels: The Sudoku puzzles progress from six grids to nine grids, with an increasing number of blank squares. | 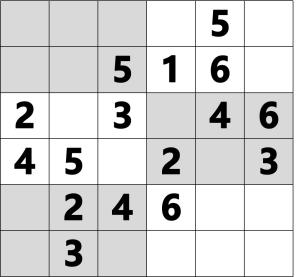 |
|  | **24 Game:** Participants use the four numbers provided in the question to perform addition, subtraction, multiplication, and division operations, aiming for the result of 24. Each number must be used exactly once.  Difficulty levels: Increase the complexity of the operations based on training progress. | 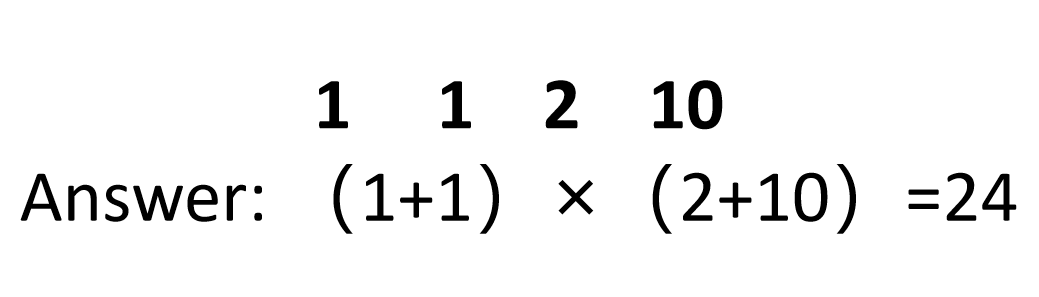 |
|  | **Matchstick training:** Participants move matchsticks around or change their quantity to create the required equation or pattern as per the question.  Difficulty levels: Increase the complexity of the equations or patterns based on training progress. | 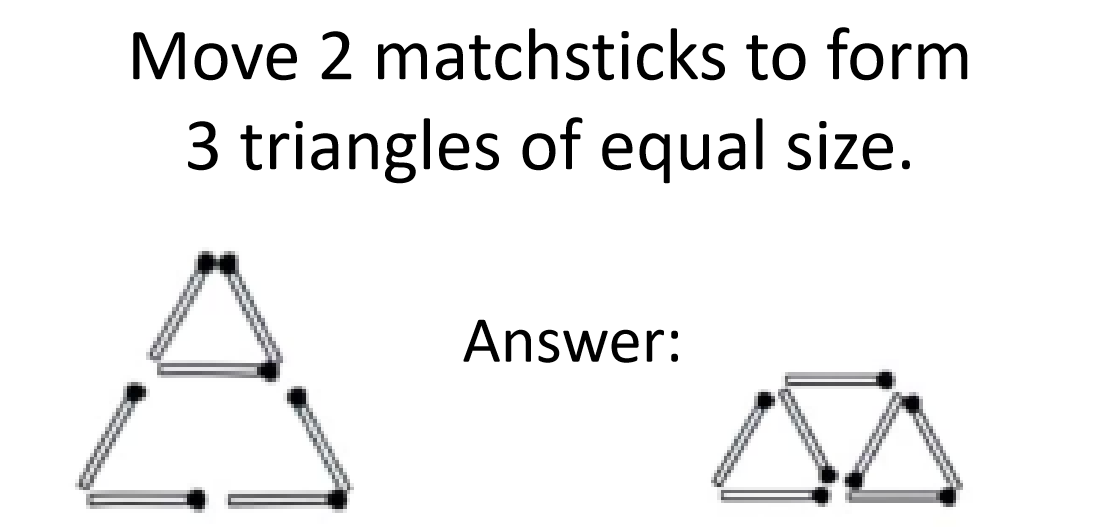 |

**Supplementary Table 1** Scheme of cognitive training (continued)

| Domain of cognition | Specific contents | Examples |
| --- | --- | --- |
| **Executive function** | **Finger exercises:** Staff members give commands, and participants follow by performing finger exercises (e.g., tapping the five fingers of both hands one by one, rubbing the thumb against the four fingers, crossing hands to pinch the nose and ears, and swapping pairs of scissors, paper, and rock gestures, etc.), each exercise repeated at least 10 times.  Difficulty levels: Increase the number of training repetitions and complexity of training exercises based on training progress. | 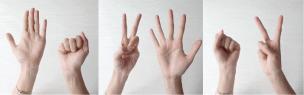 |
|  | **Building blocks/Tangram puzzles:** Participants arrange blocks/tangram pieces to match the colors and shapes shown in the workbook.  Difficulty levels: Patterns transition from regular to irregular, and the number of required blocks/tangram pieces gradually increases based on training progress. | 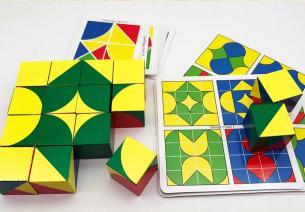 |

**Supplementary Table 2** Scheme of exercise training

| Domain of exercise | Specific contents | Examples |
| --- | --- | --- |
| **Aerobic exercise** | - Participants can choose suitable aerobic exercises based on their personal exercise habits and preferences, such as brisk walking, jogging, swimming, cycling, aerobics, Eight-section Brocade, or Tai Chi. - Each session of aerobic exercise should last at least 30 minutes. | 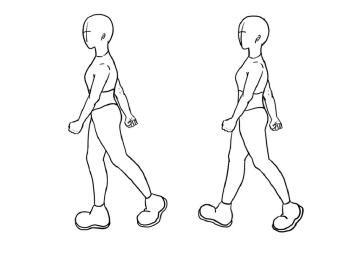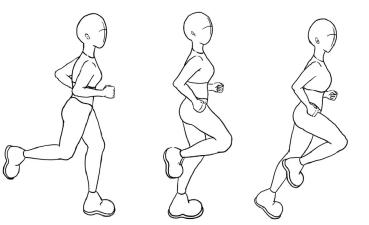 |
| **Resistance training** | **Biceps curl:**   - Sit on a sturdy chair without armrests, place the middle of a resistance band under both feet, grip the ends with palms facing up (dumbbells can be used instead of a resistance band). - Keep both upper arms as close to the sides of the body as possible, slowly bend the elbows, bringing the palms up towards the shoulders, pause briefly, then slowly return to the starting position. - Exhale while flexing the elbows, inhale while extending, maintain a slow pace; 10 reps per set, complete 2 sets. - Trained muscle: Biceps. | 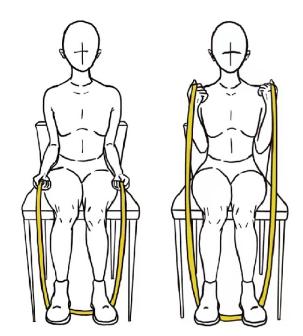 |
|  | **Resistance band row:**   - Sit on a sturdy chair without armrests, feet shoulder-width apart. - Place the middle of a resistance band under both feet, grip the ends with palms facing each other. - Lean slightly forward, relax the shoulders, keep the arms close to the sides of the body, engage the shoulder blades to pull and stretch the hands towards the armpits on both sides, pause briefly, then slowly return the hands to the starting position. - Exhale while pulling back, inhale while releasing, maintain a slow pace; 10 reps per set, complete 2 sets. - Trained muscles: Trapezius, Latissimus dorsi. | 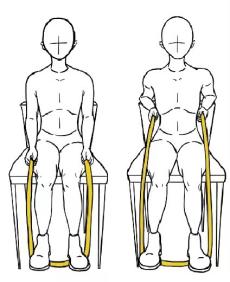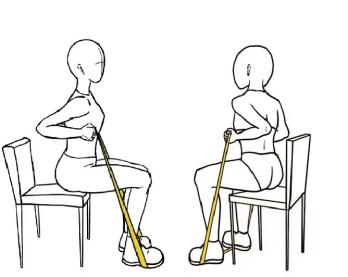 |

**Supplementary Table 2** Scheme of exercise training (continued)

| Domain of exercise | Specific contents | Examples |
| --- | --- | --- |
| **Resistance training** | **Two-arm lateral raise:**   - Stand upright with feet shoulder-width apart, knees slightly bent, hold dumbbells naturally at your sides (alternatively, step on the middle of a resistance band with both feet). - Lift your hands to shoulder height, elbows slightly bent, keeping your arms parallel to the ground, pause briefly, and slowly return both arms to the starting position. - Exhale while lifting, inhale while returning, maintain a slow pace; 10 reps per set, complete 2 sets. - Trained muscle: Deltoid. | 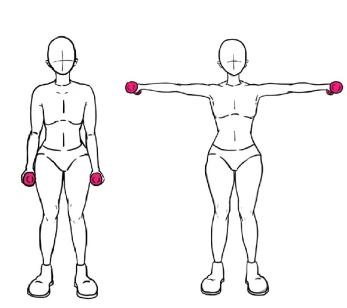 |
|  | **Leg swings forward and backward:**   - Hold onto a fixed chair or stand near a wall for stability, feet shoulder-width apart. - Try to keep the upper body upright, slowly lift your right leg forward as high as comfortable, pause briefly at the highest point, control the descent back to the starting position slowly. Then, extend the right leg backward, pause at the highest point, and slowly return to the starting position. - Exhale while lifting the leg, inhale while returning, maintain a slow pace; 10 reps per set, complete 2 sets, then switch sides. - Trained muscles: Quadriceps, Gluteus maximus. | 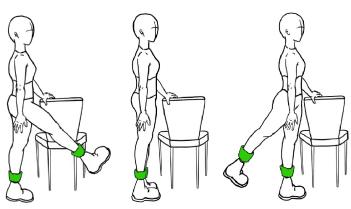 |
|  | **Hip abduction:**   - Stand upright with a sandbag or resistance band to add load to the legs, place your hands on the chair back to maintain stability. - Try to keep the upper body straight, slowly raise your right leg to the side, pause briefly at the highest point, then lower it back down slowly to the starting position. - Exhale while lifting the leg, inhale while returning, maintain a slow pace; 10 reps per set, complete 2 sets, then switch sides. - Trained muscle: Gluteus medius. | 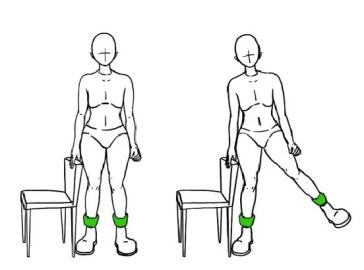 |

**Supplementary Table 2** Scheme of exercise training (continued)

| Domain of exercise | Specific contents | Examples |
| --- | --- | --- |
| **Resistance training** | **Squats:**   - Stand with feet shoulder-width apart, toes pointing slightly outward at a 30-degree angle. - Slowly bend the hips and knees, lowering your hips down, with the knees tracking over but not beyond the toes. At the same time, use your arms to maintain balance by raising them in front of you. Pause briefly when your thighs are nearly parallel to the ground, then extend the knees and hips to return to a standing position. - Inhale while squatting down, exhale while standing up, maintain a slow pace; 10 reps per set, complete 2 sets. - Trained muscles: Quadriceps, Gluteus maximus, Hamstrings. | 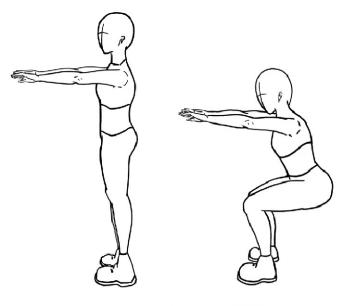 |
| **Balance training** | **Single leg stand:**   - Stand next to a sturdy chair, lift one foot to maintain balance, hold for 10~30 seconds. If necessary, place your hand on the chair to assist in balancing. - Repeat 2~4 times, then switch sides. | 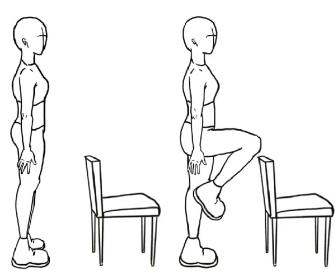 |
|  | **Calf raises:**   - Stand behind a sturdy chair or near a wall, place both hands on the chair or wall, feet shoulder-width apart. - Lift your heels off the ground, keeping your toes on the ground, raise both hands forward, hold for 10~30 seconds, then slowly return to the starting position. - Repeat 2~4 times. | 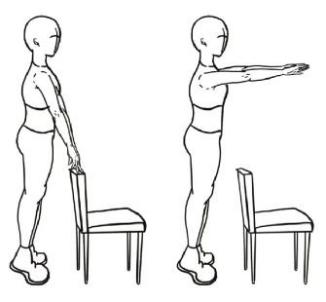 |
|  | **Walking** **heel-to-toe forward:**   - Initially, you can use the wall or another object for support. - Place the heel of your front foot directly in front of the toes of your back foot, take at least 10 steps forward, then turn around and walk back in the same manner, repeating 10 times. - Gradually transition to walking without support. | 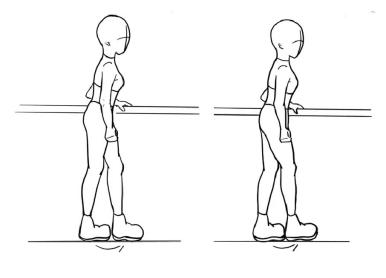 |

**Supplementary Table 2** Scheme of exercise training (continued)

| Domain of exercise | Specific contents | Examples |
| --- | --- | --- |
| **Balance training** | **Walking heel-to-toe backward:**   - Initially, you can use the wall or another object for support. - Walk backward, with the toes of the back foot close to the heel of the front foot, take at least 10 steps backward, then turn around and walk back in the same manner, repeating 10 times. - Gradually transition to walking without upper body support. | 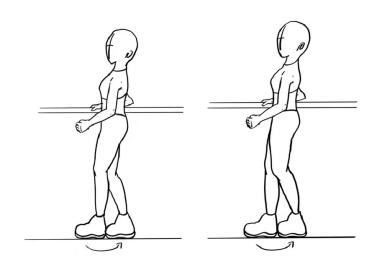 |
| **Flexibility training** | **Neck stretch:**   - Stand with feet hip-width apart, arms hanging naturally by your sides. - Gently reach down with your left arm, fingers pointing towards the floor, while tilting your head to the right so that your right ear moves towards your right shoulder, until you feel a stretch on the left side of your neck. Hold for 10~30 seconds, then switch sides. - Place your hands on your hips, tilt your head back, chin pointing towards the sky, until you feel a stretch on the front of your neck. Hold for 10~30 seconds. - Repeat 2~3 times. | 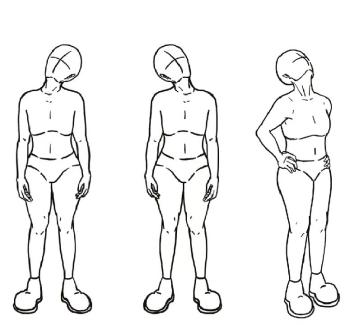 |
|  | **Back stretch:**   - Stand with feet apart, interlace your fingers and turn your palms outward as you push your hands forward. Lower your head and arch your back as far back as possible, holding the position for 10~30 seconds. Repeat 2~3 times. - Advanced (wall-assisted back stretch): Stand with your feet about 1 meter away from the wall, arms shoulder-width apart, elbows straight, palms flat against the wall. Keep your back straight, shoulders relaxed and slightly internally rotated. Lower your head between your arms, keeping your upper body straight, slowly sink your shoulders down towards the ground until they are close to parallel with the floor. - Feel the stretch in your shoulders and hold the position for 10~30 seconds. - Repeat 2~3 times. | 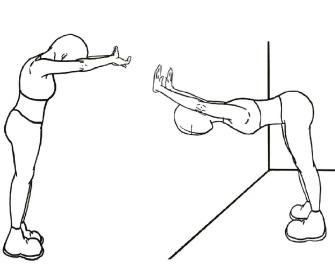 |

**Supplementary Table 2** Scheme of exercise training (continued)

| Domain of exercise | Specific contents | Examples |
| --- | --- | --- |
| **Flexibility training** | **Chest stretch:**   - Stand with feet shoulder-width apart, hands clasped behind your back with palms facing towards your body. - Slowly squeeze your shoulder blades together on both sides until you feel tension in your chest, shoulders, and arms. - Hold the position for 10~30 seconds. - Repeat 2~3 times. | 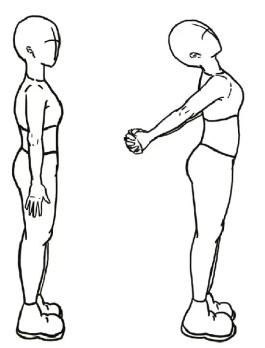 |
|  | **Thigh stretch:**   - Use a chair or wall for balance, lift your right foot and hold the right ankle with your right hand. - Tighten your abdomen, use your right hand to pull upwards, pushing your right hip forward until you feel a noticeable stretch on the front of your right thigh. - Hold the position for 10~30 seconds. - Repeat 2~3 times, then switch sides. | 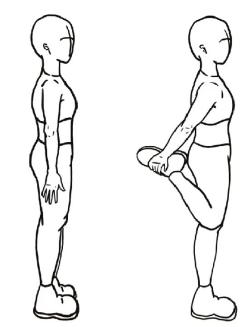 |
|  | **Calf stretch:**   - Stand facing a wall, about a step away, with feet shoulder-width apart. - Take a step forward with your right foot, bend the right knee, keep the left knee straight, point the left toes forward, and keep the left heel on the ground. Shift your weight onto your right leg by leaning your upper body forward. - Feel a slight discomfort or stretching sensation in the left calf, and hold the position for 10~30 seconds. - Repeat 2~3 times, then switch sides. | 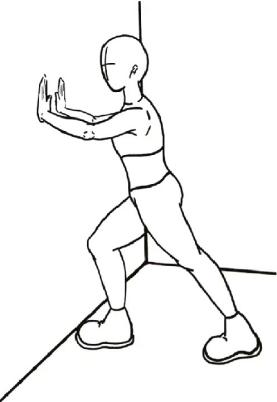 |
|  | **Hip stretch:**   - Sit on a chair with feet shoulder-width apart, bend your left knee and place your left lower leg horizontally on your right thigh, with your left ankle resting on your right knee. - Slowly lean your body forward, feeling the tension in your left hip, and hold the position for 10~30 seconds. - Repeat 2~3 times, then switch sides. | 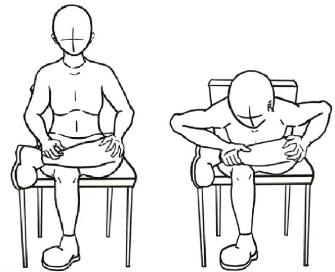 |

**Supplementary Table 3** Scheme of nutrition guidance

| Form of nutritional guidance | Specific contents | Examples |
| --- | --- | --- |
| **Dietary structure and chart** | **Dietary structure and guidelines for older adults:**  The daily diet should include staple foods (grains and tubers), vegetables and fruits, animal products (meat, poultry, fish, eggs, and dairy), and legumes.  It is recommended to consume at least 12 different types of foods daily on average and over 25 different types of foods weekly in a well-balanced manner. | 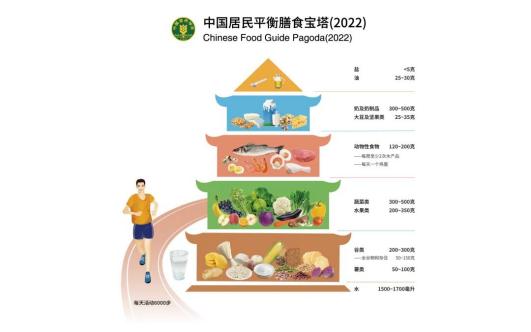 |
|  | **Food quantitative chart:**  Consume 200~300g of grain-based foods daily;  Consume more than 300g of fresh vegetables and 200~350g of fresh fruits daily;  Consume over 300mL of dairy products daily;  Consume 120~200g of animal products (meat, poultry, fish, eggs) daily;  Limit daily salt intake to less than 5g and use 25~30g of cooking oil. | 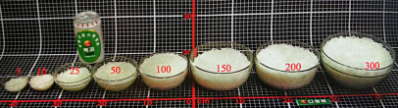 |
| **Nutritious meal demonstra-tion** | **Example 1:**  Mixed grain rice: white rice 50g + coarse grains 25g;  Stir-fried lean pork with green beans: lean meat 60g + green beans 150g ;  Tofu and vegetable soup: soft tofu 50g + greens 50g;  Cherry tomatoes: 10 pieces. | 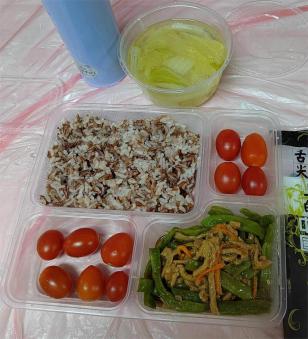 |
|  | **Example 2:**  Steamed rice: white rice 50g;  Steamed sweet potatoes: sweet potatoes 100g;  Steamed meatballs: ground lean meat 60g + tofu 50g + a little egg white and starch;  Stir-fried spinach: spinach 150g;  Tomato egg drop soup: tomatoes 100g + a little beaten egg. | 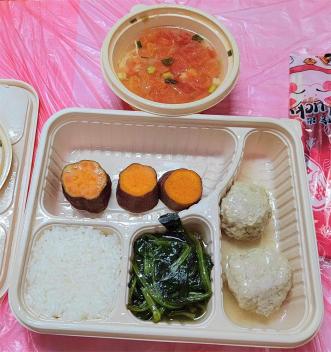 |
|  | **Example 3:**  Mixed grain rice and corn: coarse grains 50g + corn about 200g;  Stir-fried beancurd sticks, wood ear mushroom, and pork: beancurd sticks 50g + lean meat 25g + wood ear mushrooms 50g;  Mixed spring greens: spring greens 150g;  Bottle gourd and clam soup: bottle gourd 50g + a few clams | 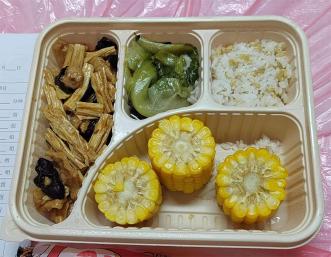 |

**Supplementary Table 3** Scheme of nutrition guidance (continued)

| Form of nutritional guidance | Specific contents |
| --- | --- |
| **Nutrition lectures** | **How to eat staple foods:**  Staple foods include rice, black rice, brown rice, oats, pearl barley, noodles, steamed buns, corn, sweet potatoes, and yams, with a recommended intake of rice 15~45g per meal (adjusted based on individual height, weight, and activity levels); choice and substitute of staple foods. |
|  | **Protein supplementation:**  It is recommended for older adults to consume 1 egg, 1 box of milk, 15~30g of lean meat or fish, and 15~30g of soy products per day; equivalent substitutions with beans and soy products. |
|  | **Diabetic diet:**  Emphasize eating at regular intervals and in controlled portions to align with the biological rhythm of insulin secretion, aiding in maintaining stable blood glucose levels; understanding foods with different glycemic indexes. |
|  | **Cardiovascular disease diet:**  Dietary principles for patients with hypertension and coronary heart disease; misconceptions in the diet of cardiovascular disease patients; selecting cooking oils and using them scientifically. |
|  | **Chronic kidney disease diet:**  Common dietary issues in chronic kidney disease; nutritional treatment principles for chronic kidney disease (low-protein diet, low-salt diet, and high-uric acid diet). |
|  | **Scientific iron supplementation:**  Older adults with poor appetite, long-term vegetarian diets, or poor digestive absorption are prone to iron deficiency; red meat, liver, and animal blood are rich sources of iron; foods rich in vitamin C such as fresh fruits and vegetables can aid in iron absorption. |
|  | **Scientific milk consumption:**  Milk is rich in calcium and protein, and it is recommended for older adults to drink 1 cup daily; differences in types of milk and how to choose; guidance for lactose intolerant individuals in selecting milk. |
|  | **What is malnutrition:**  Hazards of malnutrition in older adults; common nutrition misconceptions for older adults; signs of malnutrition and self-assessment of dietary intake; how to ensure adequate nutritional intake. |
