# Supplementary material for: Multidomain intervention for delaying aging in community-dwelling older adults (MIDA): study design and protocol
Source: Ann Med. 2025 Apr 29;57(1):2496409. doi: 10.1080/07853890.2025.2496409 (PMC12042233; doi:10.1080/07853890.2025.2496409)

SPIRIT 2013 Checklist: Recommended items to address in a clinical trial protocol and related documents*

Reported on Page

Number/Line

Number

Item

No

Reported on

Section/item

Description

Section/Paragraph

Administrative information

Descriptive title identifying the study design, population, interventions, and, if applicable, trial acronym

Trial identifier and registry name. If not yet registered, name of intended registry

All items from the World Health Organization Trial Registration Data Set

Page 1/Line 1-2

Page 6/Line 3-5

Title

Title

1

Trial registration

Study design

2a

2b

Protocol version

Funding

3

Date and version identifier

Page 6/Line 3-5 Study design

N/A N/A

N/A N/A

Sources and types of financial, material, and other support

Names, affiliations, and roles of protocol contributors

Page 27/Line 18-21

Page 1/Line 3-15

Funding

4

Roles and

5a

Authors Section

responsibilities

5b

5c

Name and contact information for the trial sponsor

Page 27/Line 7-10

Page 27/Line 7-10

Author contributions

Author contributions

Role of study sponsor and funders, if any, in study design; collection, management, analysis, and interpretation of

data; writing of the report; and the decision to submit the report for publication, including whether they will have

ultimate authority over any of these activities

5d

Composition, roles, and responsibilities of the coordinating centre, steering committee, endpoint adjudication

committee, data management team, and other individuals or groups overseeing the trial, if applicable (see Item 21a

for data monitoring committee)

N/A

N/A

Introduction

Background and

rationale

6a

Description of research question and justification for undertaking the trial, including summary of relevant studies

(published and unpublished) examining benefits and harms for each intervention

Page 3/Line 2 - Page 5/Line Introduction

17

6b

7

Explanation for choice of comparators

Specific objectives or hypotheses

Page 3/Line 2 - Page 5/Line Introduction

17

Objectives

Page5/Line 12-17

Introduction


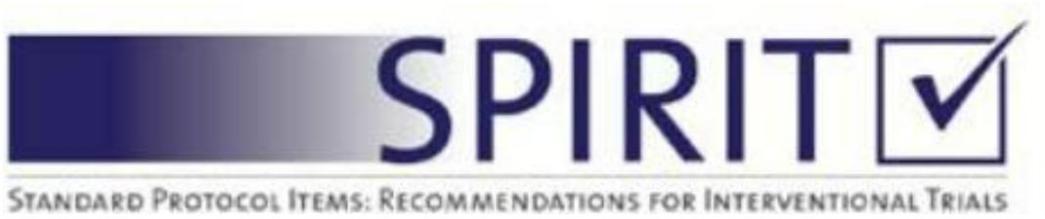

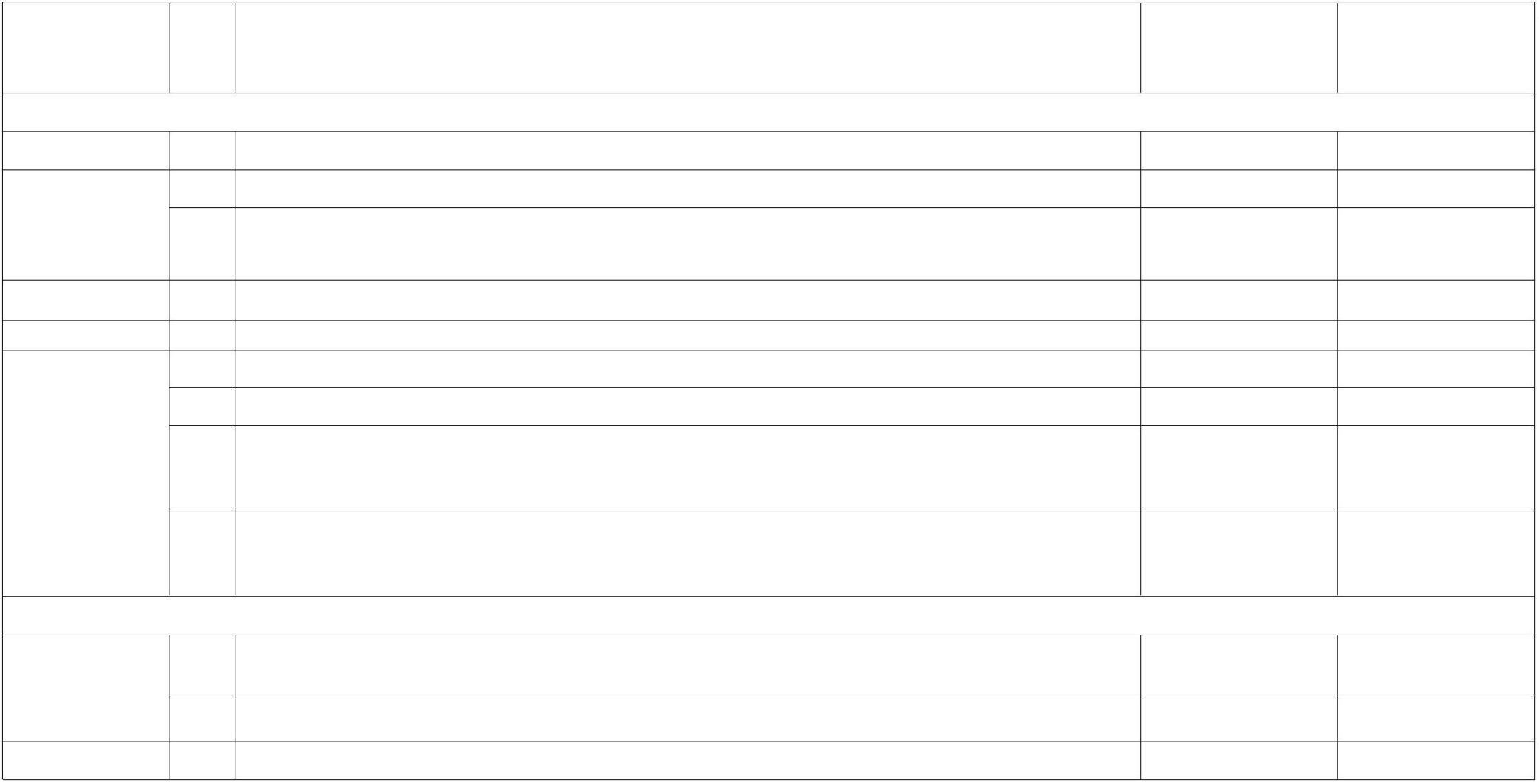


Trial design

Description of trial design including type of trial (eg, parallel group, crossover, factorial, single group), allocation ratio,

and framework (eg, superiority, equivalence, noninferiority, exploratory)

Study design

Study design

8

Page 6/Line 3-5

Methods: Participants, interventions, and outcomes

Study setting

9

Description of study settings (eg, community clinic, academic hospital) and list of countries where data will be

collected. Reference to where list of study sites can be obtained

Page 6/Line 3-5

Eligibility criteria

10

11a

Inclusion and exclusion criteria for participants. If applicable, eligibility criteria for study centres and individuals who

will perform the interventions (eg, surgeons, psychotherapists)

Page 6/Line 15-28 Inclusion and

Exclusion criteria

Interventions

Interventions for each group with sufficient detail to allow replication, including how and when they will be

administered

Page 8/Line 8 - 19 Intervention group

11b

11c

Criteria for discontinuing or modifying allocated interventions for a given trial participant (eg, drug dose change in

response to harms, participant request, or improving/worsening disease)

Page 21/Line 20-24

Safety assessment

Strategies to improve adherence to intervention protocols, and any procedures for monitoring adherence (eg, drug

tablet return, laboratory tests)

Page 6/Line 8-13 Participants and recruitment;

Page 7/Line 16-28

Randomization, blinding, and allocation concealment

Relevant concomitant care and interventions that are permitted or prohibited during the trial

Intervention group

11d

12

Page 8/Line 8-19

Outcomes

Primary, secondary, and other outcomes, including the specific measurement variable (eg, systolic blood pressure),

analysis metric (eg, change from baseline, final value, time to event), method of aggregation (eg, median, proportion),

and time point for each outcome. Explanation of the clinical relevance of chosen efficacy and harm outcomes is

strongly recommended

Outcome measures

Page 12/Line 13-29

Participant

timeline

13

14

15

Time schedule of enrolment, interventions (including any run-ins and washouts), assessments, and visits for

participants. A schematic diagram is highly recommended (see Figure)

Table 1

Table 1

Sample size

Estimated number of participants needed to achieve study objectives and how it was determined, including clinical

and statistical assumptions supporting any sample size calculations

Calculation of sample size

Page 7/Line 2 -14

Strategies for achieving adequate participant enrolment to reach target sample size

Page 7/Line 16-28 Randomization, blinding,

and allocation concealment

Methods: Assignment of interventions (for controlled trials)

Allocation:

Sequence

generation

16a

16b

Method of generating the allocation sequence (eg, computer-generated random numbers), and list of any factors for

stratification. To reduce predictability of a random sequence, details of any planned restriction (eg, blocking) should

be provided in a separate document that is unavailable to those who enrol participants or assign interventions

Page 7/Line 16-28 Randomization, blinding,

and allocation concealment

Randomization, blinding, and allocation concealment

Allocation

Mechanism of implementing the allocation sequence (eg, central telephone; sequentially numbered, opaque, sealed

envelopes), describing any steps to conceal the sequence until interventions are assigned

Page 7/Line 16-28

concealment

mechanism


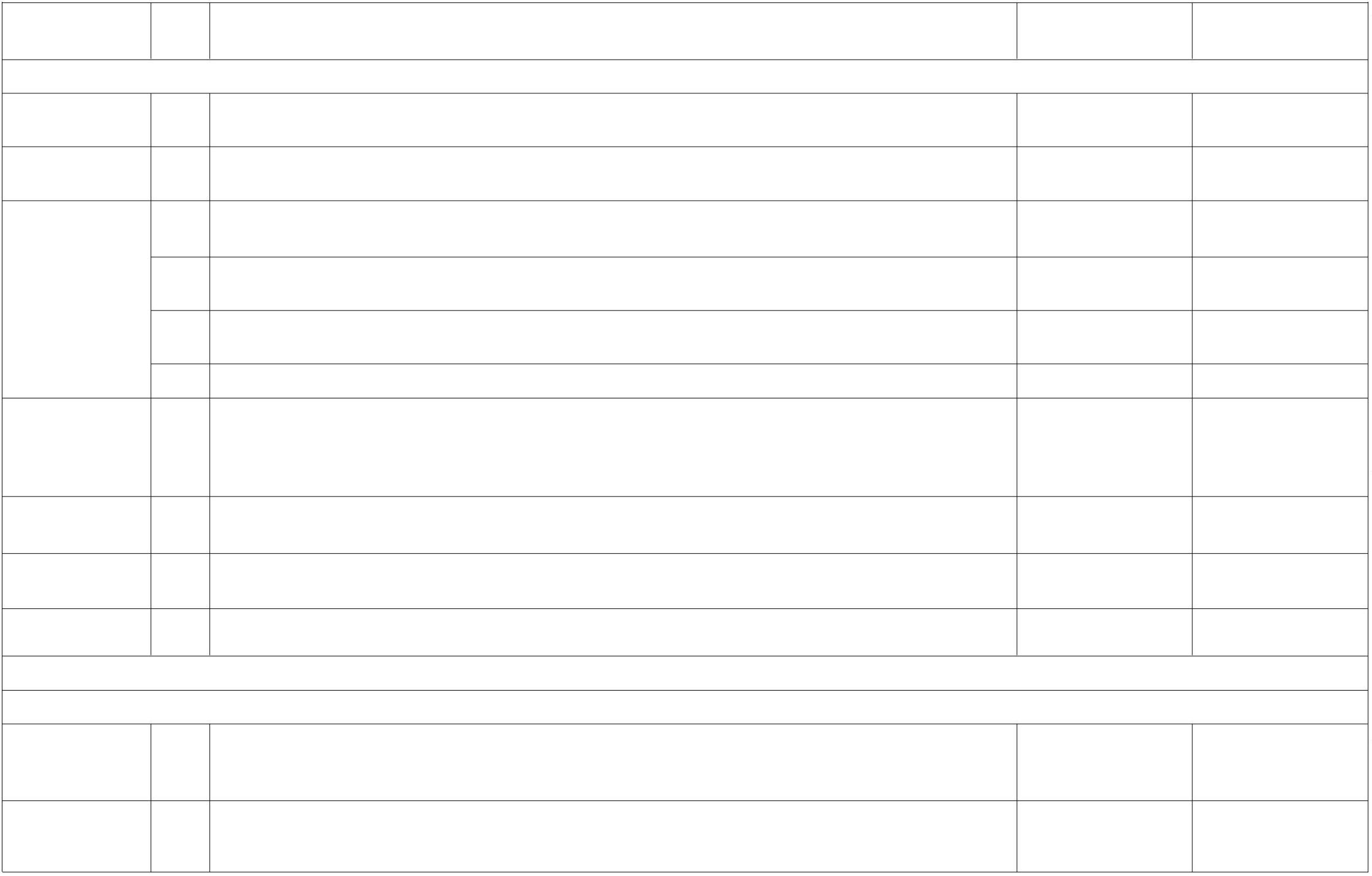


Implementation

16c

Who will generate the allocation sequence, who will enrol participants, and who will assign participants to

interventions

Randomization, blinding, and

Allocation concealment

Page 7/Line 16- 28

Blinding (masking)

Who will be blinded after assignment to interventions (eg, trial participants, care providers, outcome assessors, data

analysts), and how

17a

17b

Page 7/Line 16- 28

Page 7/Line 16-28 Randomization, blinding, and allocation concealment

Randomization, blinding,

and allocation concealment

If blinded, circumstances under which unblinding is permissible, and procedure for revealing a participant’s allocated

intervention during the trial

Methods: Data collection, management, and analysis

Data collection

methods

18a

Plans for assessment and collection of outcome, baseline, and other trial data, including any related processes to

promote data quality (eg, duplicate measurements, training of assessors) and a description of study instruments (eg,

questionnaires, laboratory tests) along with their reliability and validity, if known. Reference to where data collection

forms can be found, if not in the protocol

Outcome measures

Page 12/Line 13-29

18b

19

Plans to promote participant retention and complete follow-up, including list of any outcome data to be collected for

participants who discontinue or deviate from intervention protocols

Page 12/Line 13-29 Outcome measures

Data management

Plans for data entry, coding, security, and storage, including any related processes to promote data quality (eg,

double data entry; range checks for data values). Reference to where details of data management procedures can be

found, if not in the protocol

N/A

N/A

Statistical

methods

20a

20b

20c

Statistical methods for analysing primary and secondary outcomes. Reference to where other details of the statistical

analysis plan can be found, if not in the protocol

Statistical analysis

N/A

Page 21/ Line 2-18

Methods for any additional analyses (eg, subgroup and adjusted analyses)

N/A

Definition of analysis population relating to protocol non-adherence (eg, as randomised analysis), and any statistical

methods to handle missing data (eg, multiple imputation)

Page 21/Line 2-18

Statistical analysis

Methods: Monitoring

Data monitoring

Composition of data monitoring committee (DMC); summary of its role and reporting structure; statement of whether

it is independent from the sponsor and competing interests; and reference to where further details about its charter

can be found, if not in the protocol. Alternatively, an explanation of why a DMC is not needed

21a

N/A

N/A

N/A

21b

22

Description of any interim analyses and stopping guidelines, including who will have access to these interim results

and make the final decision to terminate the trial

N/A

Harms

Plans for collecting, assessing, reporting, and managing solicited and spontaneously reported adverse events and

other unintended effects of trial interventions or trial conduct

Page 21/Line 20-24

Safety assessment

N/A

Auditing

23

Frequency and procedures for auditing trial conduct, if any, and whether the process will be independent from

investigators and the sponsor

N/A


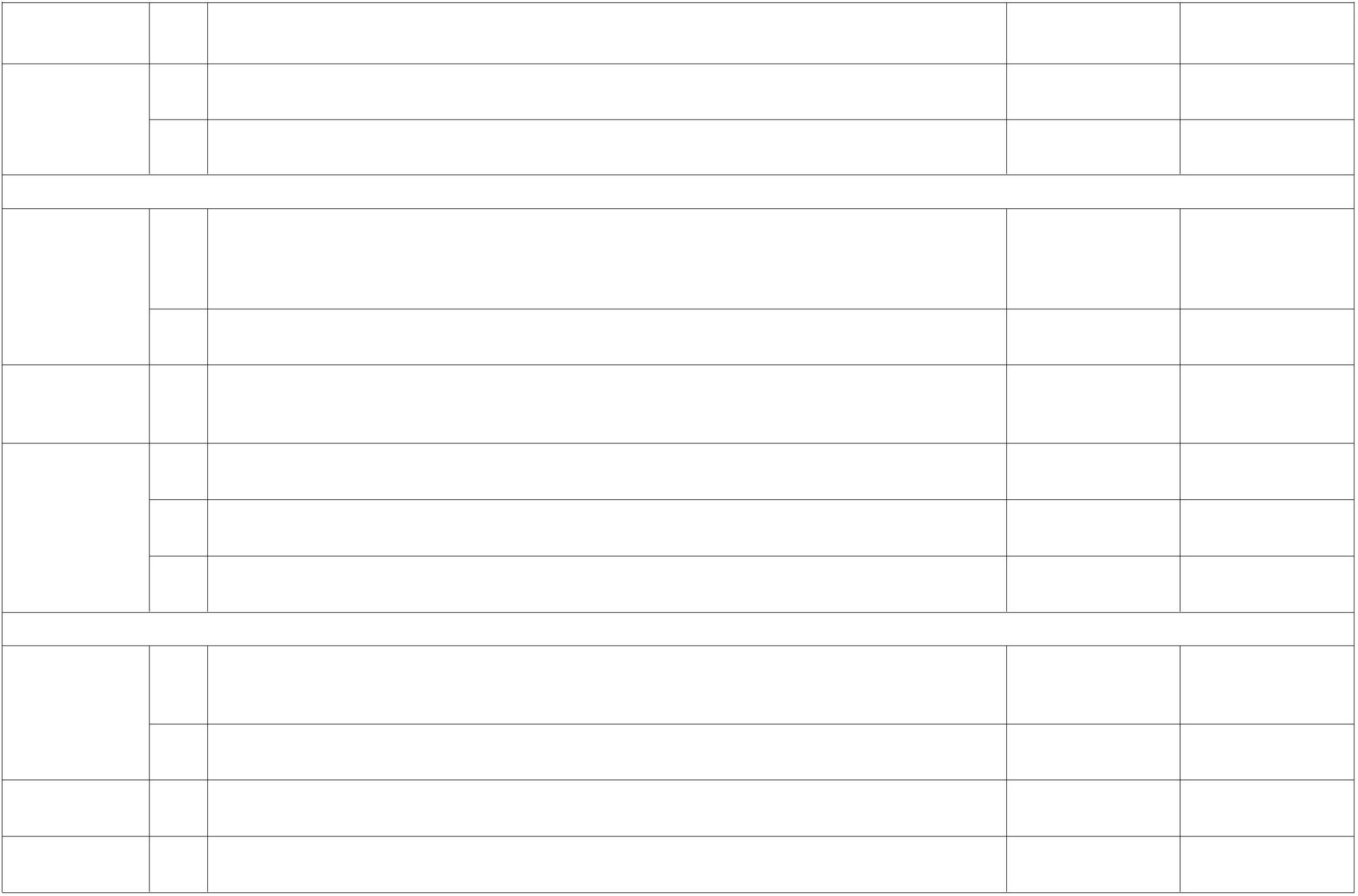


Ethics and dissemination

Research ethics

approval

24

Plans for seeking research ethics committee/institutional review board (REC/IRB) approval

Page 27/ Line 14-16

Ethical Statement

Protocol

25

Plans for communicating important protocol modifications (eg, changes to eligibility criteria, outcomes, analyses) to

relevant parties (eg, investigators, REC/IRBs, trial participants, trial registries, journals, regulators)

N/A

N/A

amendments

Consent or assent

26a

Who will obtain informed consent or assent from potential trial participants or authorised surrogates, and how (see Item 32)

Randomization, blinding, and allocation concealment

Page 7/Line 16-28

26b

27

Additional consent provisions for collection and use of participant data and biological specimens in ancillary studies,

if applicable

N/A

N/A

Confidentiality

How personal information about potential and enrolled participants will be collected, shared, and maintained in order

to protect confidentiality before, during, and after the trial

N/A

N/A

Declaration of

interests

28

29

Financial and other competing interests for principal investigators for the overall trial and each study site

Page 27/Line 18-21

Funding

Access to data

Statement of who will have access to the final trial dataset, and disclosure of contractual agreements that limit such

access for investigators

N/A

N/A

Ancillary and

post-trial care

Provisions, if any, for ancillary and post-trial care, and for compensation to those who suffer harm from trial

participation

30

Page 21/Line 20-24

Safety assessment

N/A

Dissemination

policy

31a

Plans for investigators and sponsor to communicate trial results to participants, healthcare professionals, the public,

and other relevant groups (eg, via publication, reporting in results databases, or other data sharing arrangements),

including any publication restrictions

N/A

31b

31c

Authorship eligibility guidelines and any intended use of professional writers

N/A

N/A

N/A

N/A

Plans, if any, for granting public access to the full protocol, participant-level dataset, and statistical code

Appendices

Informed consent

materials

32

33

Model consent form and other related documentation given to participants and authorised surrogates

N/A

Page 20/Line 11-19

N/A

Blood tests

Biological

specimens

Plans for collection, laboratory evaluation, and storage of biological specimens for genetic or molecular analysis in

the current trial and for future use in ancillary studies, if applicable

*It is strongly recommended that this checklist be read in conjunction with the SPIRIT 2013 Explanation & Elaboration for important clarification on the items. Amendments to the protocol should be

tracked and dated. The SPIRIT checklist is copyrighted by the SPIRIT Group under the Creative Commons “[Attribution-NonCommercial-NoDerivs 3.0 Unported](http://www.creativecommons.org/licenses/by-nc-nd/3.0/)” license.


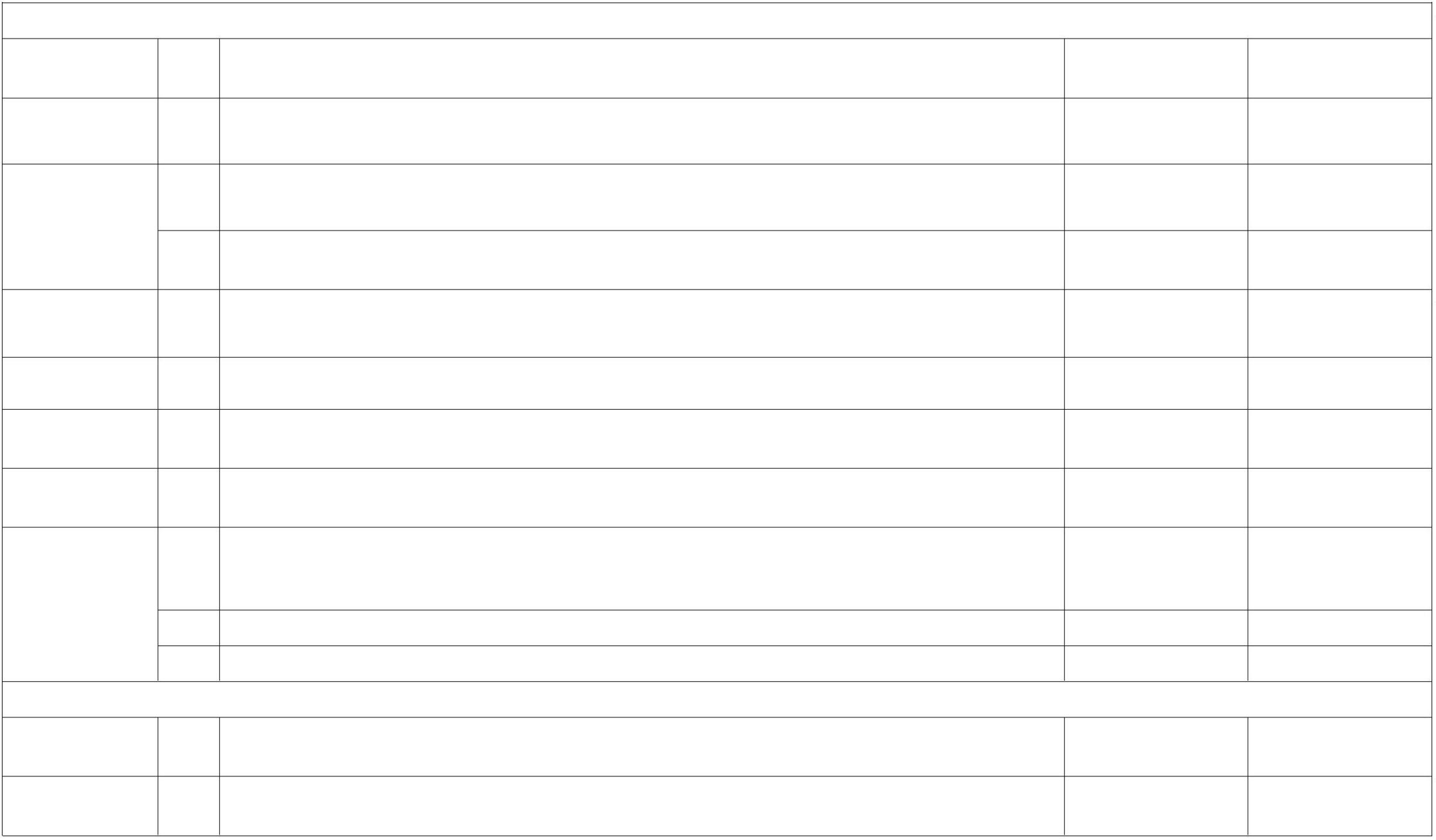

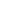

Supplement: Supplemental Material [file IANN_A_2496409_SM4210.zip › Suppl_Data/SPIRIT revised.docx]
